# Supplementary material for: DONSON is required for CMG helicase assembly in the mammalian cell cycle
Source: EMBO Rep. 2023 Oct 2;24(11):e57677. doi: 10.15252/embr.202357677 (PMC10626419; doi:10.15252/embr.202357677)
Supplement: Supplementary file 1 — Appendix [file EMBR-24-e57677-s002.pdf]

Page 1: Appendix Table of Contents

Page 2: Appendix Figure S1

Design of 5’ and 3’ homology fragments for assembly of donor vectors for C-terminal tagging in mammalian cells.

Page 3: Appendix Figure S2

Design of 5’ and 3’ homology fragments for assembly of donor vectors for N-terminal tagging in mammalian cells.

Page 4: Appendix Table S1

Reagents and resources used in this study.

Page 16: Appendix Table S2

Vectors containing tags for use in donor vectors.

Page 18: Appendix Table S3

Plasmids containing *E.coli* markers and origin to construct tagging vectors.

Design 5’ homology and 3’ homology fragments as described at bottom of page.

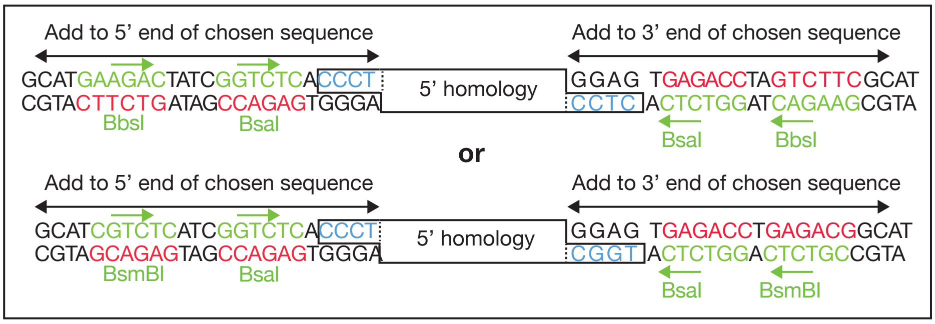

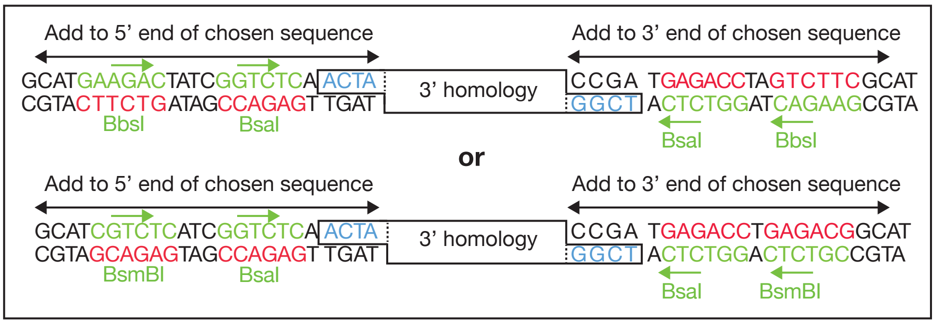

Clone each homology fragment into an entry vector (pYTK001 or pYTK001_2), using either BbsI or BsmBI type IIS enzymes. Cloned fragments will be flanked by BsaI sites. cleavage of which would leave cohesive ends for Golden Gate assembly (**5’ homology:** CCCT on 5’ side and GGAG on 3’ side; **3’ homology:** ACTA on 5’ side and CCGA on 3’ side;).

From Appendix Table S2, choose a vector with the required ‘C-terminal tag & resistance gene’, flanked by BsaI sites that will leave cohesive ends for Golden Gate assembly (GGAG on 5‘ side and ACTA on 3’ side).

From Appendix Table S3, choose a vector containing an *E. coli* selection marker and replication origin (‘Type 8’ parts from ‘Yeast Toolkit’, Lee et al, 2015), flanked by BsaI sites that will leave cohesive ends for Golden Gate assembly (CCGA on 5’ side and CCCT on 3’ side).

Assemble a new donor vector, by mixing four vectors containing: 5’ homology + 3’ homology + C-terminal tag and resistance marker + backbone. Use Golden Gate cloning with **BsaI** type IIS enzyme and T4 ligase.

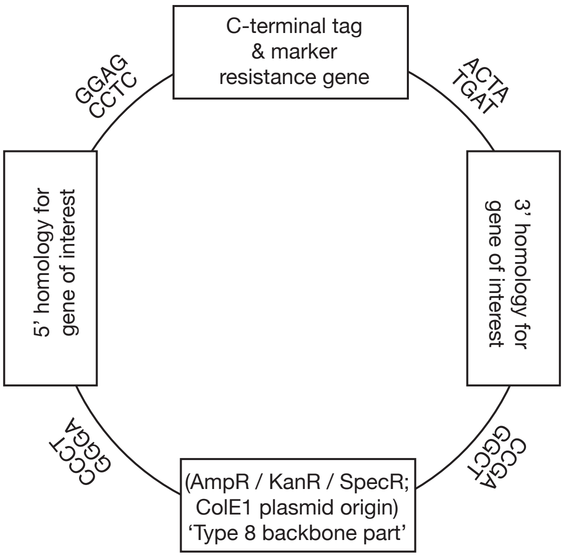

Appendix Figure S1. Design of 5’ & 3’ homology fragments for assembly of donor vectors for C-terminal tagging in mammalian cells.
For 5‘ homology fragments, select ~800bp sequence immediately upstream of STOP (do not include STOP).
For 3‘ homology fragments, select ~800bp sequence immediately downstream of STOP (do not include STOP).
Choose either BsmBI or BbsI for cloning into entry vector (pYTK001 or pYTK001_2).
If necessary, mutate any BsmBI or BbsI sites, using conservative substitiutions where necessary, to avoid changing codons.
Mutate any BsaI sites, using conservative substitiutions where necessary, to avoid changing codons.
Include mutations that will prevent binding of gRNAs used for genome editing (focus on 3’ end & PAM) to prevent re-cutting after integration.
Flank the resulting homology fragments with the sequences shown above, to allow Golden Gate assembly into entry vectors and final plasmid.

Design 5’ homology and 3’ homology fragments as described at bottom of page.

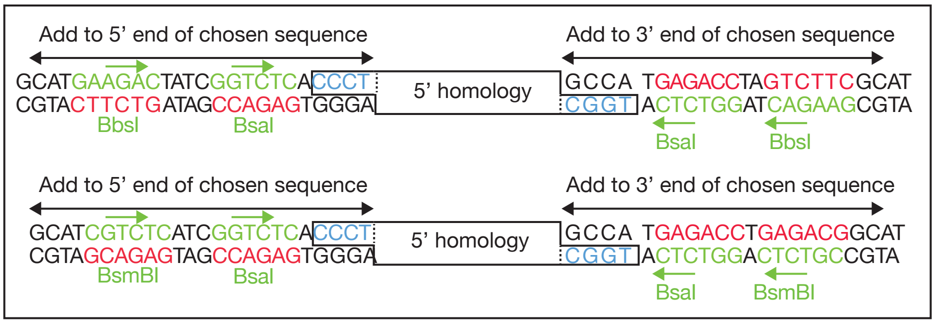

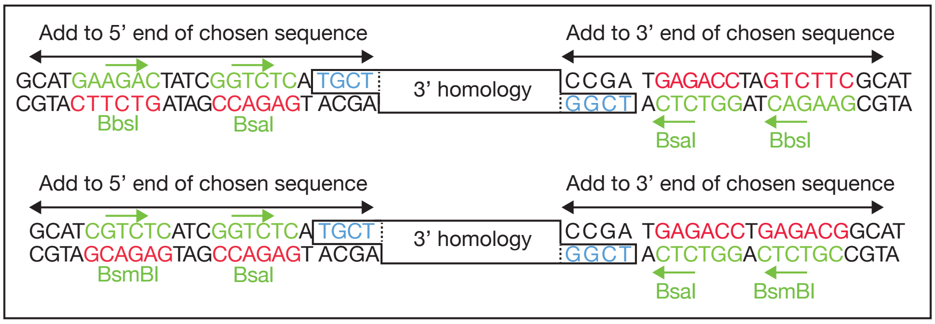

Clone each homology fragment into an entry vector (pYTK001 or pYTK001_2), using either BbsI or BsmBI type IIS enzymes.
Cloned fragments will be flanked by BsaI sites. cleavage of which would leave cohesive ends for Golden Gate assembly
(**5’ homology:** CCCT on 5’ side and GCCA on 3’ side; **3’ homology:** TGCT on 5’ side and CCGA on 3’ side;).

From Appendix Table S2, choose a vector with the required ‘N-terminal tag & resistance gene’,
flanked by BsaI sites that will leave cohesive ends for Golden Gate assembly
(GCCA on 5‘ side and TGCT on 3’ side).

From Appendix Table S3, choose a vector containing an *E. coli* selection marker and replication origin
(‘Type 8’ parts from ‘Yeast Toolkit’, Lee et al, 2015),
flanked by BsaI sites that will leave cohesive ends for Golden Gate assembly
(CCGA on 5’ side and CCCT on 3’ side).

Assemble a new donor vector, by mixing four vectors containing:
5’ homology + 3’ homology + N-terminal tag and resistance marker + backbone.
Use Golden Gate cloning with **BsaI** type IIS enzyme and T4 ligase.

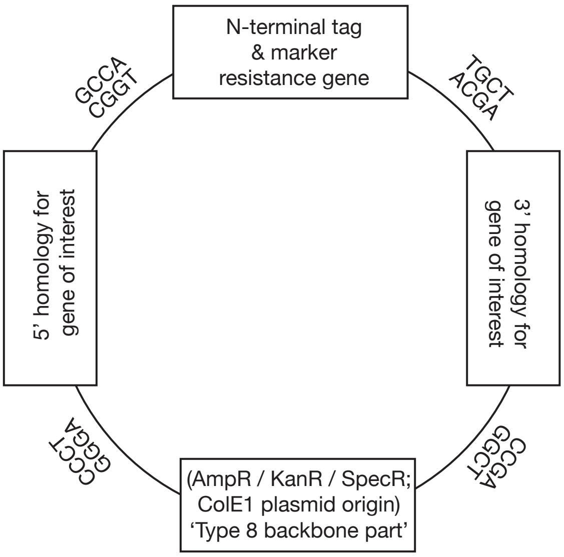

Appendix Figure S2. Design of 5’ & 3’ homology fragments for assembly of donor vectors for N-terminal tagging in mammalian cells.
For 5‘ homology fragments, select ~800bp sequence immediately upstream of initiator ATG (do not include ATG).
For 3‘ homology fragments, select ~800bp sequence immediately downstream of initiator ATG (do not include ATG).
Choose either BsmBI or BbsI for cloning into entry vector (pYTK001 or pYTK001_2).
If necessary, mutate any BsmBI or BbsI sites, using conservative substitiutions where necessary, to avoid changing codons.
Mutate any BsaI sites, using conservative substitiutions where necessary, to avoid changing codons.
Include mutations that will prevent binding of gRNAs used for genome editing (focus on 3’ end & PAM) to prevent re-cutting after integration.
Flank the resulting homology fragments with the sequences shown above, to allow Golden Gate assembly into entry vectors and final plasmid.

Appendix Table S1

Reagents and resources used in this study.

REAGENT or RESOURCE	SOURCE	IDENTIFIER
**Antibodies**		
Mouse MCM2 [antigen 1-222; sheep polyclonal]<br>Use 1 in 5,000 for immunoblotting	MRC PPU Reagents and Services<br>(Villa et al, 2021)	DU24572
Mouse MCM3 [antigen 1-222; sheep polyclonal]<br>Use 1 in 1,000 for immunoblotting	MRC PPU Reagents and Services<br>(Villa et al, 2021)	DU51847
Mouse MCM4 [antigen 1-222; sheep polyclonal]<br>Use 1 in 3,000 for immunoblotting	MRC PPU Reagents and Services<br>(Villa et al, 2021)	DU51810
Mouse MCM6 [antigen 1-222; sheep polyclonal]<br>Use 1 in 3,000 for immunoblotting	MRC PPU Reagents and Services<br>(Villa et al, 2021)	DU51793
Human MCM7 [antigen 1-719; sheep polyclonal]<br>Use 1 in 500 for immunoblotting	Santa Cruz Biotechnology	sc-9966
Mouse PSF1 [antigen 1-196; sheep polyclonal]<br>Use 1 in 3,000 for immunoblotting	MRC PPU Reagents and Services<br>(Villa et al, 2021)	DU51938
Mouse PSF3 [antigen 1-216; sheep polyclonal]<br>Use 1 in 1,000 for immunoblotting	MRC PPU Reagents and Services<br>(Villa et al, 2021)	DU24601
Mouse SLD5 [antigen 1-223; sheep polyclonal]<br>Use 1 in 2,000 for immunoblotting	MRC PPU Reagents and Services<br>(Villa et al, 2021)	DU24572
Mouse CDC45 [antigen 1-222; sheep polyclonal]<br>Use 1 in 1,000 for immunoblotting	MRC PPU Reagents and Services<br>(Villa et al, 2021)	DU35753
Mouse DONSON [antigen 1-222; sheep polyclonal]<br>Use 1 in 1,000 for immunoblotting	MRC PPU Reagents and Services	DU62042
Mouse DONSON [antigen 340-560; sheep polyclonal]<br>Use 1 in 2,000 for immunoblotting	MRC PPU Reagents and Services	DU70265

Mouse POLE1 [antigen 1-222; sheep polyclonal]<br>Use 1 in 1,000 for immunoblotting	MRC PPU Reagents and Services<br>(Villa et al, 2021)	DU27959
Mouse TIPIN [antigen 1-222; sheep polyclonal]<br>Use 1 in 1,000 for immunoblotting	MRC PPU Reagents and Services<br>(Villa et al, 2021)	DU56426
Human MCM2 (mouse monoclonal)<br>Use 1 in 1,000 for immunofluorescence	BD-Biosciences	610701
Human CDC45 (rabbit monoclonal)	Cell Signaling Technology	11881
anti-sheep IgG HRP [from donkey]<br>Use 1 in 10,000 for immunoblotting	Sigma-Aldrich	A3415
anti-mouse IgG HRP [from goat]<br>Use 1 in 3,000 for immunoblotting	Sigma-Aldrich	A4416
Anti-Mouse IgG (from donkey) conjugated to Alexa Fluor 488	Invitrogen	A-21202
Anti-Rabbit IgG (from donkey) conjugated to Alexa Fluor 488	Invitrogen	A-21206
**Chemicals, Peptides, Recombinant Proteins, other reagents**		
LIF	MRC PPU Reagents and Services	DU1715
Ubiquitin PrG	MRC PPU Reagents and Services	DU49003
Cis-AGB1	Tocris	7687
AGB1	Tocris	7686
CB-5083	Selleckchem	S8101
S-Trityl-L-cysteine (STLC)	Sigma-Aldrich	164739
Thymidine	Sigma-Aldrich	T9250
Gibson Assembly Cloning Kit	New England Biolabs	E2611
XpressRef Universal Total human RNA	QIAGEN	338112
PrimeScript™ RT-PCR Kit	TaKaRa	RR014
T4 polynucleotide kinase	New England Biolabs	M201
T4 DNA ligase	New England Biolabs	M202
PrimeStar Hot Start DNA polymerase	Takara Bio	R010A
Ubiquitin PrG	MRC PPU Reagents and Services	DU49003

Pierce Universal Nuclease	ThermoFisher Scientific	88702
Gelatin	Sigma-Aldrich	G1890
DMEM	ThermoFisher Scientific	11960044
'No phenol red DMEM'	ThermoFisher Scientific	21063029
Knockout serum replacement	ThermoFisher Scientific	10828028
FBS	LabTech	FCS-SA/500
PEI	Polysciences, Inc	24765-2
L-Glutamine	ThermoFisher Scientific	25030081
Penicillin-Streptomycin	ThermoFisher Scientific	15140122
Sodium Pyruvate	ThermoFisher Scientific	11360070
non-essential amino acids	ThermoFisher Scientific	11140050
β-mercaptoethanol	Sigma-Aldrich	M6520
Trypsin-EDTA	ThermoFisher Scientific	25300054
Puromycin	ThermoFisher Scientific	A1113802
Hygromycin	Invitrogen	ant-hg-5
Blasticidin	Invitrogen	ant-bl-10p
RNase A	ThermoFisher Scientific	EN0531
Propidium iodide	Sigma-Aldrich	P4170
DAPI	Thermo	62248
Crystal violet	Sigma-Aldrich	HT90132
Ni-NTA agarose	Qiagen	30210
Protein A/G Sepharose beads	Expedeon	AGA1000
Dynabeads M-270 Epoxy	Life Technologies	14302D
TEV protease	MRC PPU Reagents and Services	DU6811
ULP1 HIS-SENP1 (415-647)	MRC PPU Reagents and Services	DU39129
Roche cOmplete EDTA-free protease inhibitor cocktail	Roche	11873580001
5-ethynyl-2'-deoxyuridine (EdU)	Thermo Fisher Scientific Inc.	E10187
Click-iT EdU Imaging Kit	Thermo Fisher Scientific Inc.	B10184

Cell culture plates, 96-well, CELLSTAR with μClear bottom	Greiner	655090
μ-Slide 4-well	Ibidi	80426
Yeast Extract	Becton Dickinson	2127750
Peptone	Oxoid	LP0037B
Raffinose	Formedium	RAF04
Galactose	Formedium	GAL03
StrataClone Blunt PCR cloning kit	Agilent Technologies	240207
4-12% Bis-Tris NuPAGE	Life Technologies	NP0301
iBlot membrane	ThermoFisher Scientific	IB301031
Simply Blue	ThermoFisher Scientific	LC6060
Amicon Ultra-15 centrifugal Filter Unit, 30kDa	Millipore	UFC903024
Amicon Ultra-15 centrifugal Filter Unit, 100kDa	Millipore	UFC910024
ECL western blotting detection reagent	VWR	RPN2106
4X LDS sample buffer	ThermoFisher Scientific	NP0007
**Experimental Models: Organisms/Strains**		
E14tg2A mouse ES cells		N/A
E14tg2A *TAP-SLD5*	Villa et al, 2021	N/A
E14tg2A *TAP-DONSON*	This study	N/A
E14tg2A *DONSON-TAP*	This study	N/A
E14tg2A *TAP-PSF1*	This study	N/A
E14tg2A *Bromo-Tag-DONSON*	This study	N/A
E14tg2A *TAP-SLD5 Bromo-Tag-DONSON*	This study	N/A
E14tg2A *TAP-PSF1 Bromo-Tag-DONSON*	This study	N/A
E14tg2A *GFP-SLD5*	Villa et al, 2021	N/A
E14tg2A *GFP-SLD5 Bromo-Tag-DONSON*	This study	N/A
E14tg2A *Bromo-Tag-PSF1*	This study	N/A
E14tg2A *mCherry-PSF1 DONSON-GFP*	This study	N/A
Saccharomyces cerevisiae: YJF1 *MAT***a** *ade2-1 ura3-1 his3-11,15 trp1-1 leu2-3,112 can1-100, pep4Δ::kanMX , bar1Δ::hphNT*	Frigola et al., 2013	N/A
Saccharomyces cerevisiae: YCE923 *MATα ade2-1 ura3-1 his3-11,15 trp1-1 leu2-3,112 can1-100 bar1Δ::hphNT*	This study	N/A
Saccharomyces cerevisiae: YCE1206 *MATα ade2-1 ura3-1 his3-11,15 trp1-1 leu2-3,112 can1-100 bar1Δ::hphNT leu2Δ::pCE207(MCM2-GAL1,10-MCM3; MCM4-GAL1,10-MCM6; MCM5-GAL1,10-MCM7)*	This study	N/A

Saccharomyces cerevisiae: YCE1259 (to purify mouse CMG):<br>*MAT***a** *ade2-1 ura3-1 his3-11,15 trp1-1 leu2-3,112 can1-100, pep4Δ::kanMX , bar1Δ::hph-NT*<br>*leu2::MCM2-GAL1,10-MCM3- MCM4-GAL1,10-MCM6- MCM5-GAL1,10-MCM7*<br>*HO:: PSF1-GAL1,10-PSF2- PSF3-GAL1,10-ProteinA-SLD5- CDC45-GAL1,10-spacer*	This study	N/A
**Oligonucleotides**		
Mouse DONSON Exon 1 gRNA1_oligo 1, 9552: caccgACAGCCATTGCGCAGCACCG	This study	N/A
Mouse DONSON Exon 1 gRNA1_oligo 2, 9553: aaacCGGTGCTGCGCAATGGCTGTc	This study	N/A
Mouse DONSON Exon 1 gRNA2_oligo 1, 9554: CACCGGCTACTCGCCTAGCTTCAAG	This study	N/A
Mouse DONSON Exon 1 gRNA2_oligo 2, 9555: AAACCTTGAAGCTAGGCGAGTAGCC	This study	N/A
Mouse DONSON Exon 4 gRNA1_oligo 1, 9560: caccgCGCTCATTAAAGCATGCTGC	This study	N/A
Mouse DONSON Exon 4 gRNA1_oligo 2, 9561: aaacGCAGCATGCTTTAATGAGCGc	This study	N/A
Mouse DONSON Exon 4 gRNA2_oligo 1, 9562: caccgGGTAAGCTAGCCCAGTCCAG	This study	N/A
Mouse DONSON Exon 4 gRNA2_oligo 2, 9563: aaacCTGGACTGGGCTAGCTTACCc	This study	N/A
Mouse DONSON Exon 10 gRNA1_oligo 1, 9581: caccgTTAGGCTTACTTTGGTGTTC	This study	N/A
Mouse DONSON Exon 10 gRNA1_oligo 2, 9582: aaacGAACACCAAAGTAAGCCTAAc	This study	N/A
Mouse DONSON Exon 10 gRNA2_oligo 1, 9583: caccgGAGGAAAAAGCTTCCTAGCA	This study	N/A
Mouse DONSON Exon 10 gRNA2_oligo 2, 9584: aaacTGCTAGGAAGCTTTTTCCTCc	This study	N/A

Mouse PSF1 Exon 1 gRNA1_oligo 1, 9774: caccgCCGCGAGTTACACCGCGCGC	This study	N/A
Mouse PSF1 Exon 1 gRNA1_oligo 2, 9775: aaacGCGCGCGGTGTAACTCGCGGc	This study	N/A
Mouse PSF1 Exon 1 gRNA2_oligo 1, 9776: caccgAGAACATGACGGACGCGCCC	This study	N/A
Mouse PSF1 Exon 1gRNA2_oligo 2, 9777: aaacGGGCGCGTCCGTCATGTTCTc	This study	N/A
Mouse DONSON Exon 1 PCR forward primer, 9564: CACCGGGAAATCTGTTGGG	This study	N/A
Mouse DONSON Exon 1 PCR reverse primer, 9565: CCTAAGCGATGAGGGAATGTG	This study	N/A
Mouse DONSON Exon 10 PCR forward primer, 9587: GCATCCTAAAACCCTGGAGC	This study	N/A
Mouse DONSON Exon 10 PCR reverse primer, 9594: CATACGTGAGAGCAGAGGAACTAG	This study	N/A
Mouse PSF1 PCR forward primer, 9782: CGAGGAGCTGGTTGCTGGTG	This study	N/A
Mouse PSF1 PCR reverse primer, 9795: GCAACAGCAGGCATAGGCTC	This study	N/A
Mouse DONSON forward primer for construction of pCE302, 9503: GAACAGATTGGTGGCATGGCTGTCTCGGTGCCCGGC	This study	N/A
Mouse DONSON reverse primer for construction of pCE302, 9504: GTGCGGCCGCTTATTAGGATCTCCAACTTAAAATGTAGTCACTC	This study	N/A
M13 Forward: GTAAAACGACGGCCAGT		
M13 reverse: GGAAACAGCTATGACCATG		
**Recombinant DNA (Plasmids)**		
pX335 [expression vector for gRNA and Cas9-D10A 'nickase']	Pyzocha et al, 2014	Addgene
pKN7 [expression vector for gRNA and Puromycin-resistance gene]	MRC PPU Reagents and Services	DU70250
pJA5 [gRNA1 DONSON Exon 1]	MRC PPU Reagents and Services (This study)	DU75972

pJA6 [gRNA2 DONSON Exon 1]	MRC PPU Reagents and Services (This study)	DU75973
pJA11 [gRNA3 DONSON Exon 4]	MRC PPU Reagents and Services (This study)	DU75974
pJA12 [gRNA4 DONSON Exon4]	MRC PPU Reagents and Services (This study)	DU75975
pCE308 [gRNA1 DONSON Exon 10]	MRC PPU Reagents and Services (This study)	DU75976
pCE309 [gRNA2 DONSON Exon 10]	MRC PPU Reagents and Services (This study)	DU75977
pCE319 [gRNA1 PSF1 Exon 1]	MRC PPU Reagents and Services (This study)	DU75978
pCE320 [gRNA2 PSF1 Exon 1]	MRC PPU Reagents and Services (This study)	DU75979
pCE310 [donor vector TAP-DONSON]	MRC PPU Reagents and Services (This study)	DU75980
pCE361 [donor vector DONSON-TAP]	MRC PPU Reagents and Services (This study)	DU75981
pCE318 [donor vector TAP-PSF1]	MRC PPU Reagents and Services (This study)	DU75982
p3481 [donor vector Bromotag-DONSON - Hygromycin]	MRC PPU Reagents and Services (This study)	DU75983
pCE335 [donor vector Bromotag-DONSON - Blasticidin]	MRC PPU Reagents and Services (This study)	DU75984

pCE334 [donor vector mCherry-PSF1]	MRC PPU Reagents and Services (This study)	DU78025
pCE333 [donor vector DONSON-GFP]	MRC PPU Reagents and Services (This study)	DU75985
pCE340 [donor vector Bromotag-PSF1]	MRC PPU Reagents and Services (This study)	DU75986
pCE302 [bacterial expression of Mouse DONSON with 14His-Smt3 in pK27SUMO vector]	MRC PPU Reagents and Services (This study)	DU75987
pCE207 [yeast expression of Mouse MCM2-7]	MRC PPU Reagents and Services (This study)	DU78032
pCE297 [yeast expression of Mouse CDC45 and GINS]	MRC PPU Reagents and Services (This study)	DU75988
pYTK001 [GFP entry vector for yeast and mammalian toolkits]	Lee et al (2015)	Addgene (#65108)
pYTK001_2 [RFP entry vector for 'mammalian toolkit']	MRC PPU Reagents and Services (This study)	DU75989
pYTK83 [AmpR+ColE1 'type 8 backbone part']	Lee et al (2015)	Addgene (#65190)
pYTK84 [KanR+ColE1 'type 8 backbone part']	Lee et al (2015)	Addgene (#65191)
pYTK85 [SpecR+ColE1 'type 8 backbone part']	Lee et al (2015)	Addgene (#65192)
pCE342 [for plasmid assembly via 'mammalian toolkit': N-terminal tagging with BSD-P2A-BromoTag cassette]	MRC PPU Reagents and Services (This study)	DU75990
pCE348 [for plasmid assembly via 'mammalian toolkit': N-terminal tagging with BSD-P2A-BromoTag-GFP cassette]	MRC PPU Reagents and Services (This study)	DU75991
pCE349 [for plasmid assembly via 'mammalian toolkit': N-terminal tagging with BSD-P2A-BromoTag-mCherry cassette]	MRC PPU Reagents and Services (This study)	DU75992

pCE343 [for plasmid assembly via 'mammalian toolkit': N-terminal tagging with HygR-P2A-BromoTag cassette]	MRC PPU Reagents and Services (This study)	DU75993
pCE350 [for plasmid assembly via 'mammalian toolkit': N-terminal tagging with HygR-P2A-BromoTag-GFP cassette]	MRC PPU Reagents and Services (This study)	DU78026
pCE351 [for plasmid assembly via 'mammalian toolkit': N-terminal tagging with HygR-P2A-BromoTag-mCherry cassette]	MRC PPU Reagents and Services (This study)	DU78027
pCE352 [for plasmid assembly via 'mammalian toolkit': C-terminal tagging with BromoTag-GFP-PGKprom-BSD]	MRC PPU Reagents and Services (This study)	DU75994
pCE353 [for plasmid assembly via 'mammalian toolkit': C-terminal tagging with BromoTag-GFP-PGKprom-HygR]	MRC PPU Reagents and Services (This study)	DU78028
pCE354 [for plasmid assembly via 'mammalian toolkit': C-terminal tagging with BromoTag-mCherry-PGKprom-BSD]	MRC PPU Reagents and Services (This study)	DU78029
pCE355 [for plasmid assembly via 'mammalian toolkit': C-terminal tagging with BromoTag-mCherry-PGKprom-HygR]	MRC PPU Reagents and Services (This study)	DU75995
pCE341 [for plasmid assembly via 'mammalian toolkit': C-terminal tagging with BromoTag-PGKprom-BSD]	MRC PPU Reagents and Services (This study)	DU75996
pCE356 [for plasmid assembly via 'mammalian toolkit': C-terminal tagging with BromoTag-PGKprom-HygR]	MRC PPU Reagents and Services (This study)	DU75997
pCE357 [for plasmid assembly via 'mammalian toolkit': N-terminal tagging with BSD-P2A-GFP cassette]	MRC PPU Reagents and Services (This study)	DU75998
pCE326 [for plasmid assembly via 'mammalian toolkit': N-terminal tagging with BSD-P2A-mCherry cassette]	MRC PPU Reagents and Services (This study)	DU75999
pCE325 [for plasmid assembly via 'mammalian toolkit': N-terminal tagging with HygR-P2A-GFP cassette]	MRC PPU Reagents and Services (This study)	DU76000

pCE358 [for plasmid assembly via ‘mammalian toolkit’: N-terminal tagging with HygR-P2A-mCherry cassette]	MRC PPU Reagents and Services (This study)	DU78001
pCE359 [for plasmid assembly via ‘mammalian toolkit’: C-terminal tagging with GFP-PGKprom-BSD]	MRC PPU Reagents and Services (This study)	DU78002
pCE327 [for plasmid assembly via ‘mammalian toolkit’: C-terminal tagging with GFP-PGKprom-HygR]	MRC PPU Reagents and Services (This study)	DU78003
pCE360 [for plasmid assembly via ‘mammalian toolkit’: C-terminal tagging with mCherry-PGKprom-BSD]	MRC PPU Reagents and Services (This study)	DU78004
pCE370 [for plasmid assembly via ‘mammalian toolkit’: C-terminal tagging with mCherry-PGKprom-HygR]	MRC PPU Reagents and Services (This study)	DU78005
pCE371 [for plasmid assembly via ‘mammalian toolkit’: C-terminal tagging with TAP-PGKprom-BSD]	MRC PPU Reagents and Services (This study)	DU78006
pCE372 [for plasmid assembly via ‘mammalian toolkit’: C-terminal tagging with TAP-PGKprom-HygR]	MRC PPU Reagents and Services (This study)	DU78007
pCE315 [for plasmid assembly via ‘mammalian toolkit’: N-terminal tagging with HygR-P2A-TAP cassette]	MRC PPU Reagents and Services (This study)	DU78008
pCE344 [for plasmid assembly via ‘mammalian toolkit’: N-terminal tagging with BSD-P2A-TAP cassette]	MRC PPU Reagents and Services (This study)	DU78033
pCE373 [for plasmid assembly via ‘mammalian toolkit’: C-terminal tagging with 5FLAG-PGKprom-BSD]	MRC PPU Reagents and Services (This study)	DU78009
pCE374 [for plasmid assembly via ‘mammalian toolkit’: C-terminal tagging with 5FLAG-PGKprom-HygR]	MRC PPU Reagents and Services (This study)	DU78010
pCE375 [for plasmid assembly via ‘mammalian toolkit’: N-terminal tagging with BSD-P2A-5FLAG cassette]	MRC PPU Reagents and Services (This study)	DU78034

pCE376 [for plasmid assembly via ‘mammalian toolkit’: N-terminal tagging with HygR-P2A-5FLAG cassette]	MRC PPU Reagents and Services (This study)	DU78011
pCE377 [for plasmid assembly via ‘mammalian toolkit’: C-terminal tagging with 6HA-PGKprom-BSD]	MRC PPU Reagents and Services (This study)	DU78012
pCE378 [for plasmid assembly via ‘mammalian toolkit’: C-terminal tagging with 6HA-PGKprom-HygR]	MRC PPU Reagents and Services (This study)	DU78030
pCE379 [for plasmid assembly via ‘mammalian toolkit’: N-terminal tagging with BSD-P2A-6HA cassette]	MRC PPU Reagents and Services (This study)	DU78035
pCE380 [for plasmid assembly via ‘mammalian toolkit’: N-terminal tagging with HygR-P2A-6HA cassette]	MRC PPU Reagents and Services (This study)	DU78031
pCE381 [for plasmid assembly via ‘mammalian toolkit’: C-terminal tagging with BromoTag-dTAG-PGKprom-BSD]	MRC PPU Reagents and Services (This study)	DU78013
pCE382 [for plasmid assembly via ‘mammalian toolkit’: C-terminal tagging with BromoTag-dTAG-PGKprom-HygR]	MRC PPU Reagents and Services (This study)	DU78014
pCE383 [for plasmid assembly via ‘mammalian toolkit’: C-terminal tagging with dTAG-BromoTag-PGKprom-BSD]	MRC PPU Reagents and Services (This study)	DU78015
pCE384 [for plasmid assembly via ‘mammalian toolkit’: C-terminal tagging with dTAG-BromoTag-PGKprom-HygR]	MRC PPU Reagents and Services (This study)	DU78016
pCE385 [for plasmid assembly via ‘mammalian toolkit’: N-terminal tagging with BSD-P2A-BromoTag-dTAG cassette]	MRC PPU Reagents and Services (This study)	DU78017
pCE386 [for plasmid assembly via ‘mammalian toolkit’: N-terminal tagging with BSD-P2A-dTAG-BromoTag cassette]	MRC PPU Reagents and Services (This study)	DU78018
pCE387 [for plasmid assembly via ‘mammalian toolkit’: N-terminal tagging with HygR-P2A-BromoTag-dTAG cassette]	MRC PPU Reagents and Services (This study)	DU78019

pCE388 [for plasmid assembly via 'mammalian toolkit': N-terminal tagging with HygR-P2A-dTAG-BromoTag cassette]	MRC PPU Reagents and Services (This study)	DU78020
**Software and Algorithms**		
Prism 9	GraphPad	https://www.graphpad.com/scientific-software/prism/
FlowJo	BD Biosciences	https://www.flowjo.com/
Tableau	Tableau	https://www.tableau.com
**Other:**		
ScanR High Content Screening Microscopy (Olympus).	Thermo Fisher Scientific Inc.	N/A

Appendix Table S2

Vectors containing tags for use in donor vectors.

Vectors are described in Appendix Figures S1-S2. HygR = hygromycin resistance marker; BSD = Blastocidin resistance marker; P2A = ribosomal skipping sequence; TAP = Tandem Affinity Purification tag; GFP = Green Fluorescent Protein; mCherry = red fluorescent protein; BromoTag = degron cassette degraded in response to AGB1 PROTAC that recruits $CUL2^{VHL}$ ubiquitin ligase; 5FLAG = 5X FLAG epitope; 6HA = 6X hemagglutinin epitope; dTAG = degron cassette degraded in response to PROTACs recruiting CRBN ubiquitin ligase; PGKprom = PGK promoter sequence.

PLASMID	LOCATION OF TAG	TAG
pCE315	N-terminal	HygR-P2A-TAP
pCE325	N-terminal	HygR-P2A-GFP
pCE326	N-terminal	BSD-P2A-mCherry
pCE342	N-terminal	BSD-P2A-BromoTag
pCE343	N-terminal	HygR-P2A-BromoTag
pCE344	N-terminal	BSD-P2A-TAP
pCE348	N-terminal	BSD-P2A-BromoTag-GFP
pCE349	N-terminal	BSD-P2A-BromoTag-mCherry
pCE350	N-terminal	HygR-P2A-BromoTag-GFP
pCE351	N-terminal	HygR-P2A-BromoTag-mCherry
pCE357	N-terminal	BSD-P2A-GFP
pCE358	N-terminal	HygR-P2A-mCherry
pCE375	N-terminal	BSD-P2A-5FLAG
pCE376	N-terminal	HygR-P2A-5FLAG
pCE379	N-terminal	BSD-P2A-6HA
pCE380	N-terminal	HygR-P2A-6HA
pCE385	N-terminal	BSD-P2A-BromoTag-dTAG
pCE386	N-terminal	BSD-P2A-dTAG-BromoTag
pCE387	N-terminal	HygR-P2A-BromoTag-dTAG
pCE388	N-terminal	HygR-P2A-dTAG-BromoTag
pCE327	C-terminal	GFP-PGKprom-HygR
pCE341	C-terminal	BromoTag-PGKprom-BSD
pCE352	C-terminal	BromoTag-GFP-PGKprom-BSD
pCE353	C-terminal	BromoTag-GFP-PGKprom-HygR
pCE354	C-terminal	BromoTag-mCherry-PGKprom-BSD
pCE355	C-terminal	BromoTag-mCherry-PGKprom-HygR
pCE356	C-terminal	BromoTag-PGKprom-HygR
pCE359	C-terminal	GFP-PGKprom-BSD
pCE360	C-terminal	mCherry-PGKprom-BSD
pCE370	C-terminal	mCherry-PGKprom-HygR
pCE371	C-terminal	TAP-PGKprom-BSD
pCE372	C-terminal	TAP-PGKprom-HygR
pCE373	C-terminal	5FLAG-PGKprom-BSD
pCE374	C-terminal	5FLAG-PGKprom-HygR
pCE377	C-terminal	6HA-PGKprom-BSD

pCE378	C-terminal	6HA-PGKprom-HygR
pCE381	C-terminal	BromoTag-dTAG-PGKprom-BSD
pCE382	C-terminal	BromoTag-dTAG-PGKprom-HygR
pCE383	C-terminal	dTAG-BromoTag-PGKprom-BSD
pCE384	C-terminal	dTAG-BromoTag-PGKprom-HygR

Appendix Table S3

Plasmids containing *E.coli* markers and origin to construct tagging vectors.

These vectors correspond to 'Type 8 backbone parts' from Lee et al (2015) and can be used to assemble tagging vectors as described in Appendix Figures 1-2. The vectors below are available from Addgene (http://www.addgene.org).

**PLASMID**	***E. coli* marker**	**Origin of replication**
pYTK83	AmpR	ColE1
pYTK84	KanR	ColE1
pYTK85	SpecR	ColE1
