# Supplementary material for: DONSON is required for CMG helicase assembly in the mammalian cell cycle
Source: EMBO Rep. 2023 Oct 2;24(11):e57677. doi: 10.15252/embr.202357677 (PMC10626419; doi:10.15252/embr.202357677)
Supplement: Supplementary file 2 — Expanded View Figures PDF [file EMBR-24-e57677-s007.pdf]

Expanded View Figures

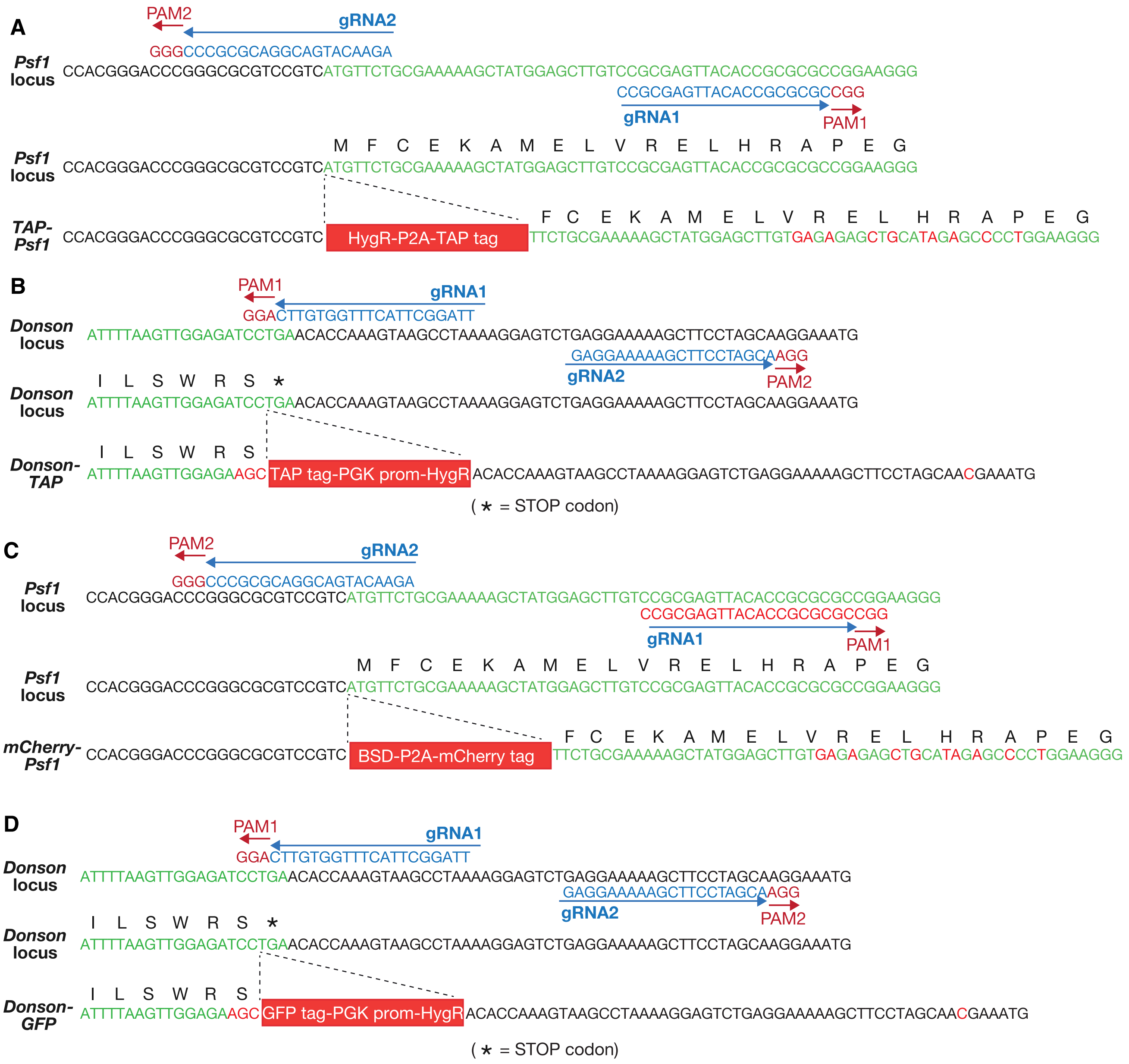

Figure EV1. Genome editing of the *Psf1* and *Donson* loci in mouse embryonic stem cells by CRISPR-Cas9-D10A.

A N-terminal tagging of PSF1 with Tandem Affinity Purification tag (TAP tag). Plasmids expressing *Psf1* gRNA1 (pCE319) and *Psf1* gRNA2 (pCE320), together with the Cas9-D10A nickase and a donor vector (pCE318), were used to introduce the indicated cassette before the initiator ATG of the *Psf1* gene. The tag comprises a Hygromycin resistance marker followed by the P2A sequence (that causes ribosomal skipping during translation) and the TAP tag. In this way, the cells express TAP-PSF1 and the Hygromycin resistance marker as separate proteins.

B C-terminal tagging of DONSON with TAP. Plasmids expressing *Donson* gRNA1 (pCE308) and *Donson* gRNA2 (pCE309), together with the Cas9-D10A nickase and the donor vector pCE361, were used to introduce the indicated cassette in place of the STOP codon at the 3′ end of the *Donson* gene. The inserted cassette comprises the TAP tag (in frame with DONSON) followed by Hygromycin resistance marker under control of the *PGK1* promotor.

C N-terminal tagging of PSF1 with mCherry. As for (A) above, except that the tag comprises the Blastocidin S deaminase gene from *Aspergillus terreus*, followed by the P2A sequence and the mCherry fluorescent protein (donor vector pCE334). In this way, the cells express mCherry-PSF1 and the Blastocidin resistance marker as separate proteins.

D C-terminal tagging of DONSON with GFP. As for (B) above but with GFP in place of TAP (donor vector pCE333).

 © 2023 The Authors

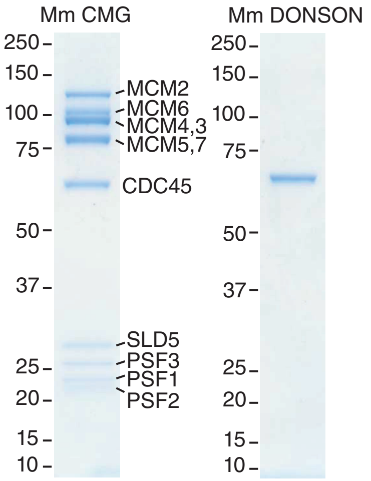

Figure EV2. Purified recombinant versions of mouse CMG and DONSON.

Mouse CMG was expressed and purified from budding yeast cells, whereas DONSON was expressed and purified from bacteria, as described in Materials and Methods. The purified proteins were resolved by SDS–PAGE and stained with colloidal Coomassie blue.

Source data are available online for this figure.

Figure EV3. Analysis of viable clones with small deletions at the beginning of the *Donson* coding sequence in mouse ES cells.

A Location of Donson gRNA1 (pJA5) and Donson gRNA2 (pJA6) are shown, together with the associated Protospacer Adjacent Motifs in the genomic sequence. Cas9-D10A cuts close to the PAM sequences and thus drives the formation of small deletions or insertions when the cut sequences are repaired.

B Immunoblot analysis of selected clones, after transfection of mouse ES cells with *Donson* gRNAs 1 + 2.

C–E DNA sequence analysis of both alleles of *Donson* in selected clones from (B).

Source data are available online for this figure.

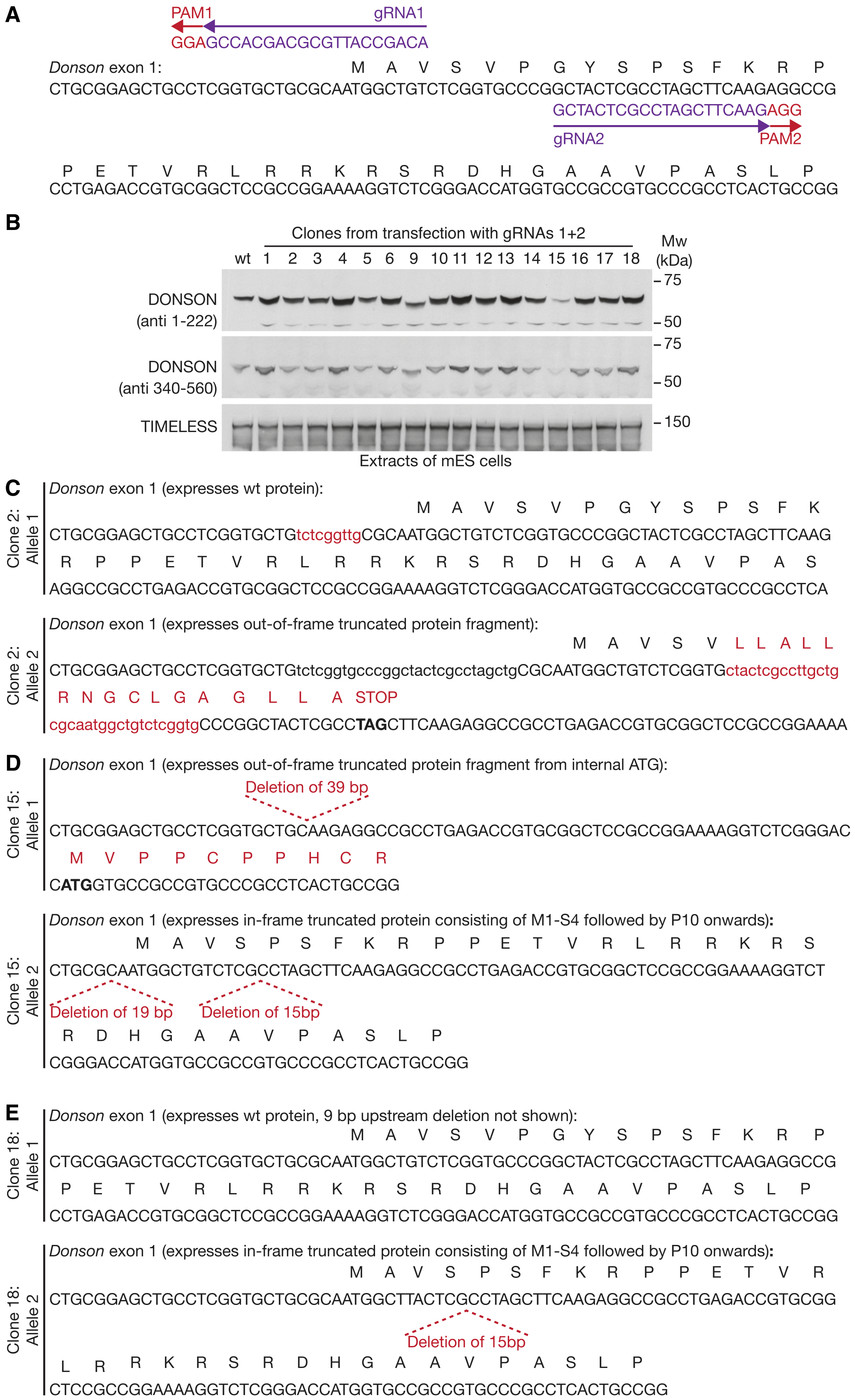

Figure EV3.

 © 2023 The Authors

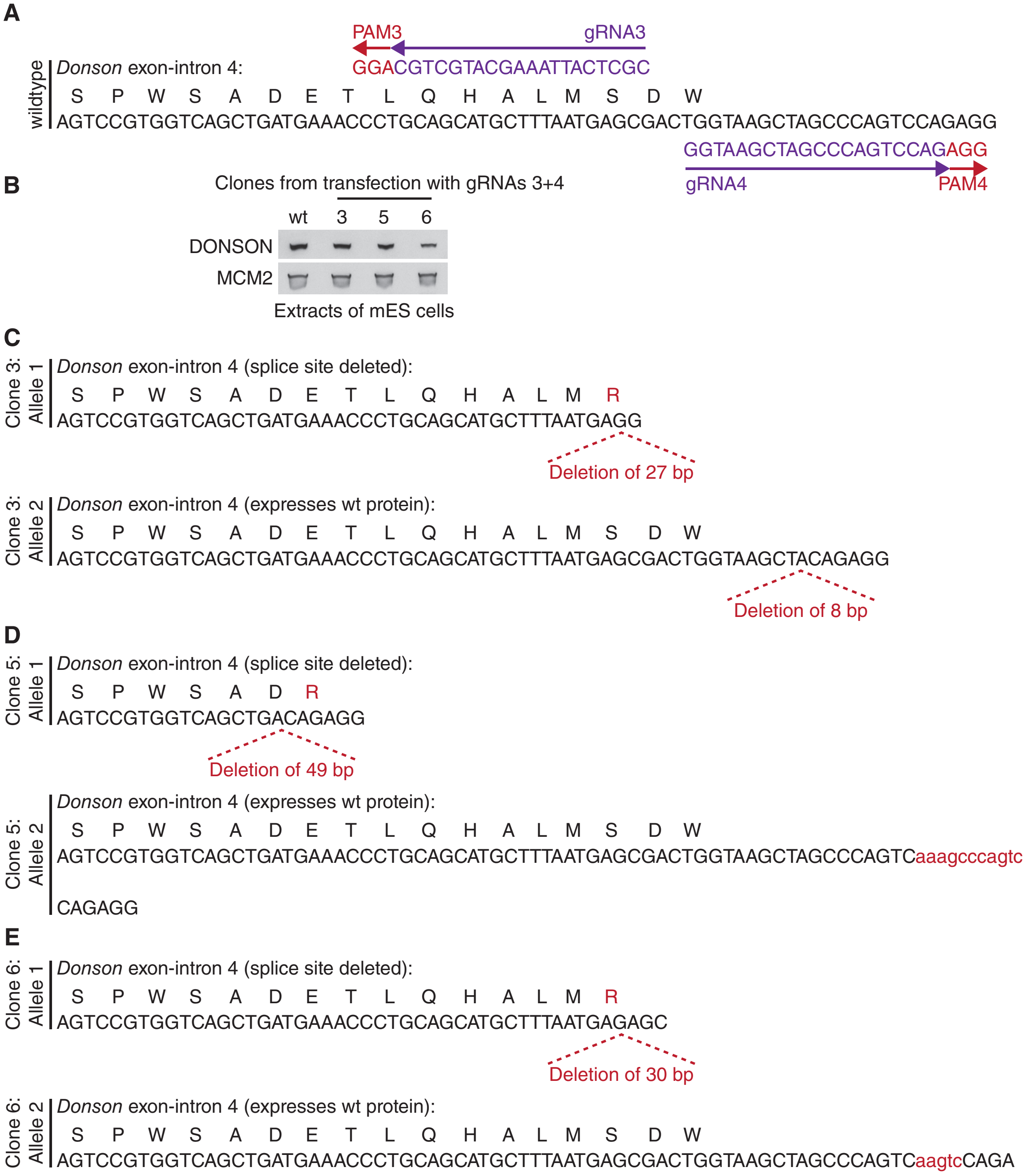

Figure EV4. Analysis of viable clones with small deletions at the end of exon 4 of the *Donson* gene in mouse ES cells.

A Location of Donson gRNA3 (pJA11) and Donson gRNA4 (pJA12) are shown, together with the associated Protospacer Adjacent Motifs in the genomic sequence. Cas9-D10A cuts close to the PAM sequences and thus drives the formation of small deletions or insertions when the cut sequences are repaired.

B Immunoblot analysis of selected clones, after transfection of mouse ES cells with *Donson* gRNAs 3 + 4.

C–E DNA sequence analysis of both alleles of *Donson* in clones from (B).

Source data are available online for this figure.

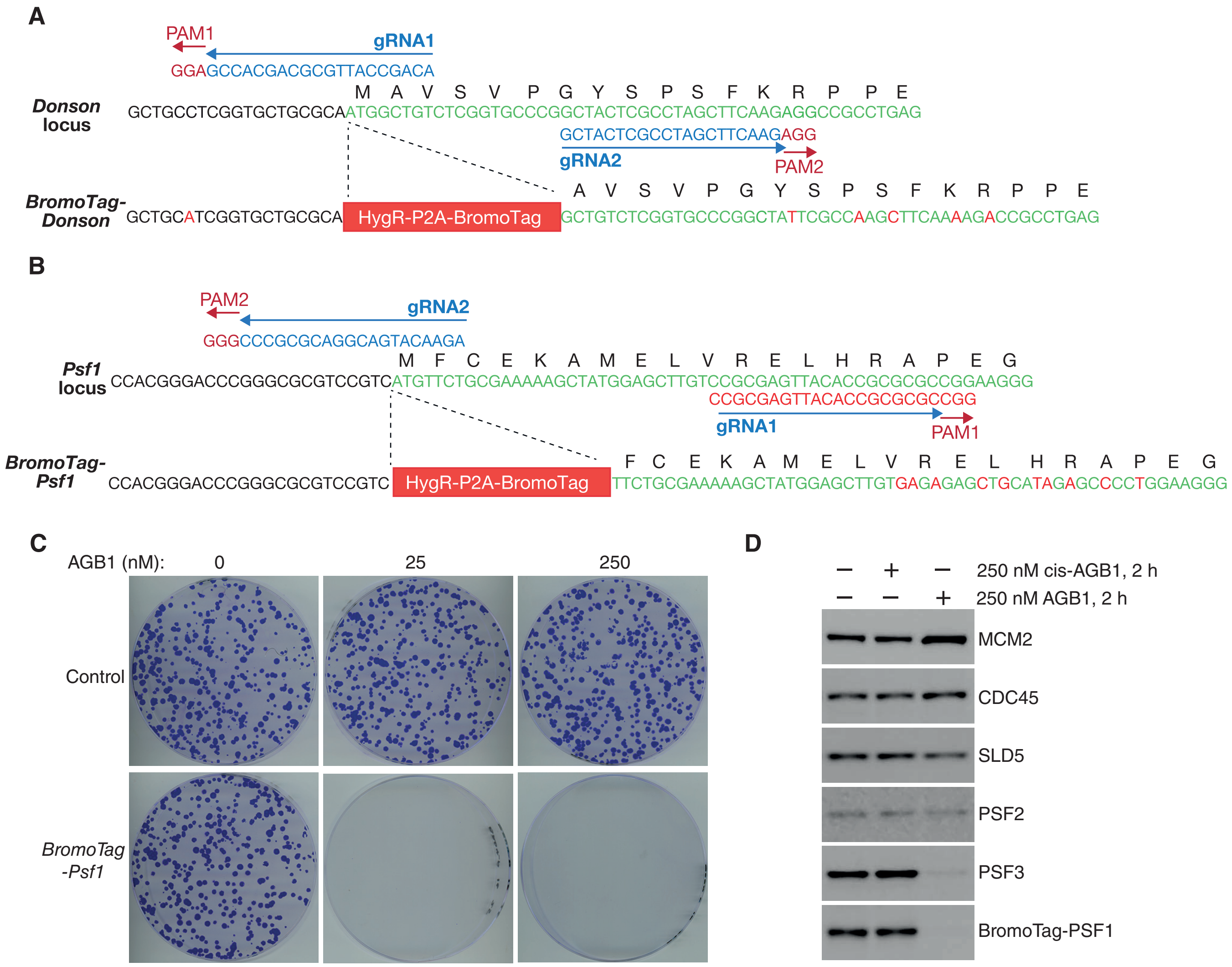

Figure EV5. Genome editing of mouse embryonic stem cells to introduce the BromoTag degron cassette into the Donson and Psf1 loci.

A N-terminal tagging of DONSON with the BromoTag degron cassette. Plasmids expressing *Donson* gRNA1 (pJA5) and *Donson* gRNA2 (pJA6), together with the Cas9-D10A nickase, were used to introduce the indicated cassette before the initiator ATG of the *Donson* gene. The tag comprises a Hygromycin resistance marker followed by the P2A sequence (that causes ribosomal skipping during translation) and the BromoTag. In this way, *BromoTag-Donson* cells express BromoTag-DONSON and the Hygromycin resistance marker as separate proteins.

B N-terminal tagging of PSF1 with the BromoTag degron cassette. Plasmids expressing *Psf1* gRNA1 (pCE319) and *Psf1* gRNA2 (pCE320), together with the Cas9-D10A nickase, were used to introduce the indicated cassette before the initiator ATG of the *Psf1* gene. *BromoTag-Psf1* cells express BromoTag-PSF1 and the Hygromycin resistance marker as separate proteins.

C Control and *BromoTag-Psf1* cells were grown for 7 days in the presence of the indicated concentrations of AGB1, before staining with crystal violet solution as described in Materials and Methods.

D Immunoblots showing the level of the indicated factors after degradation of BromoTag-PSF1, by treating cells with 250 nM AGB1 for 2 h. cis-AGB1 provides a negative control and has the *cis*- instead of *trans*-hydroxyproline group, abrogating binding to the VHL component of the ubiquitin ligase $CUL2^{VHL}$.

Source data are available online for this figure.

 © 2023 The Authors
