# Supplementary material for: DONSON is required for CMG helicase assembly in the mammalian cell cycle
Source: EMBO Rep. 2023 Oct 2;24(11):e57677. doi: 10.15252/embr.202357677 (PMC10626419; doi:10.15252/embr.202357677)
Supplement: Supplementary file 3 — Source Data for Expanded View [file EMBR-24-e57677-s001.zip › Expanded View_Source Data/EV5C/Crystal violet staining of cell colonies on plates.pdf]

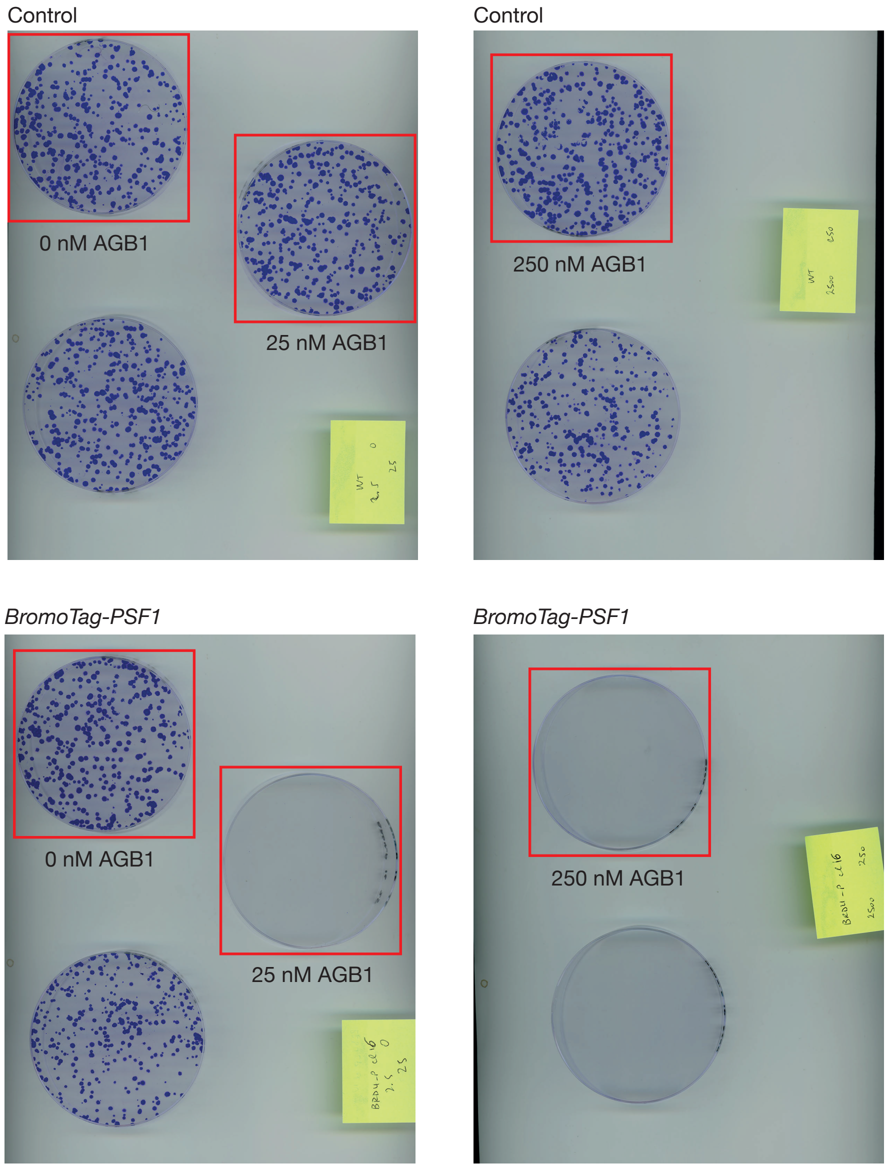

Source data for Figure EV5C.
The panels correspond illustrate scanned images of petri dishes with mouse ES cells of the indicated genotypes, treated as shown and then stained with crystal violet. Boxes indicate the areas cropped in the figure.
