# Supplementary material for: DONSON is required for CMG helicase assembly in the mammalian cell cycle
Source: EMBO Rep. 2023 Oct 2;24(11):e57677. doi: 10.15252/embr.202357677 (PMC10626419; doi:10.15252/embr.202357677)
Supplement: Supplementary file 3 — Source Data for Expanded View [file EMBR-24-e57677-s001.zip › Expanded View_Source Data/EV3B/Immunoblots for DONSON and TIMELESS.pdf]

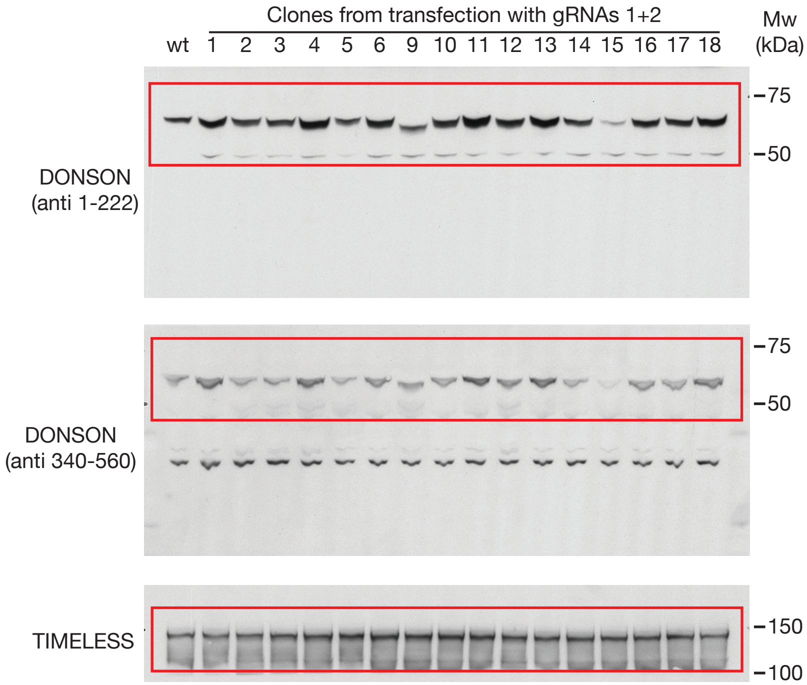

Source data for Figure EV3B.
The panels correspond to entire strips of membrane that were used for immunoblotting. Boxes indicate the areas cropped in the figure.
