# Supplementary figures and images for: DONSON is required for CMG helicase assembly in the mammalian cell cycle
Source: EMBO Rep. 2023 Oct 2;24(11):e57677. doi: 10.15252/embr.202357677 (PMC10626419; doi:10.15252/embr.202357677)

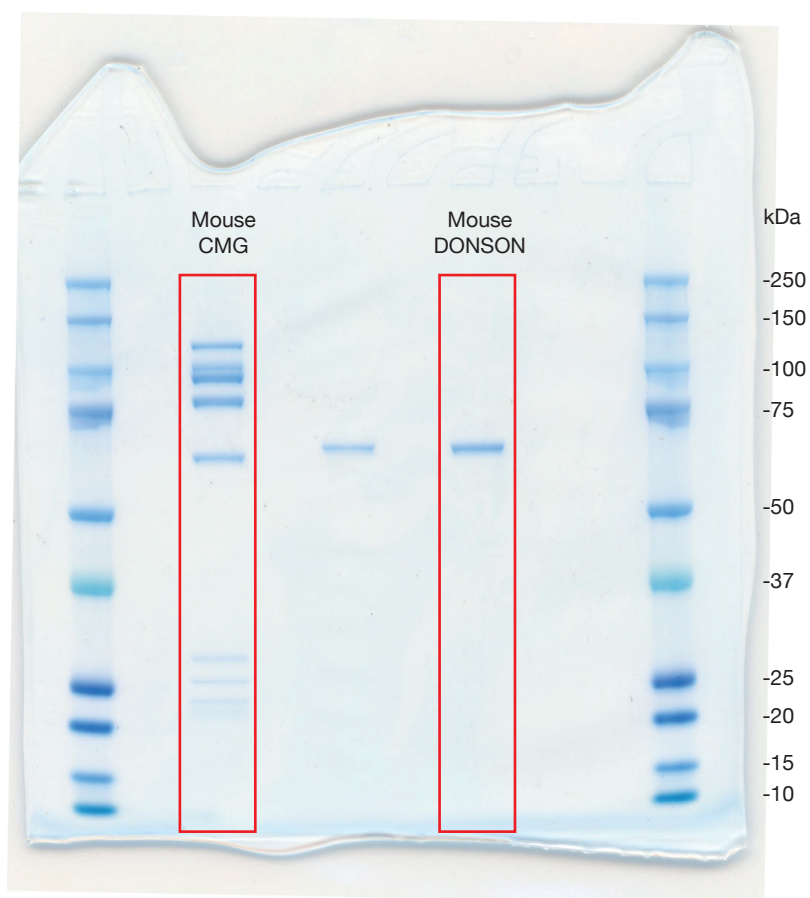

**Source data for Figure EV2.**

Coomassie-stained gel - boxes indicate the areas cropped in the figure.

Supplement: Supplementary file 3 — Source Data for Expanded View [file EMBR-24-e57677-s001.zip › Expanded View_Source Data/EV2/Coomassie stained gel.pdf]

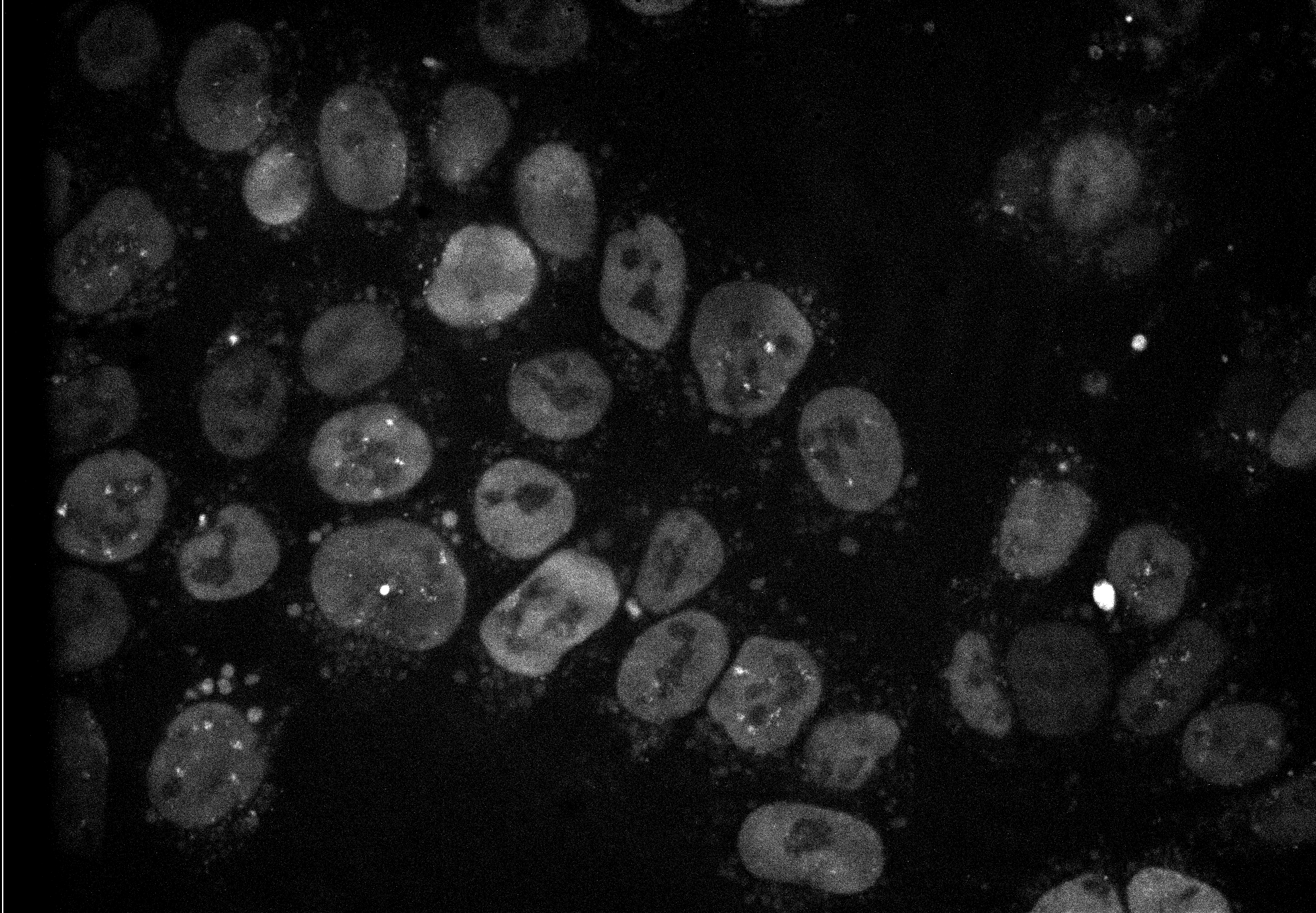

Supplement: Supplementary file 5 — Source Data for Figure 1 [file EMBR-24-e57677-s009.zip › Source data for Fig 1/EMBOR-2023-57677V2_SourceDataForFigure1D_DONSON-GFP.tif]

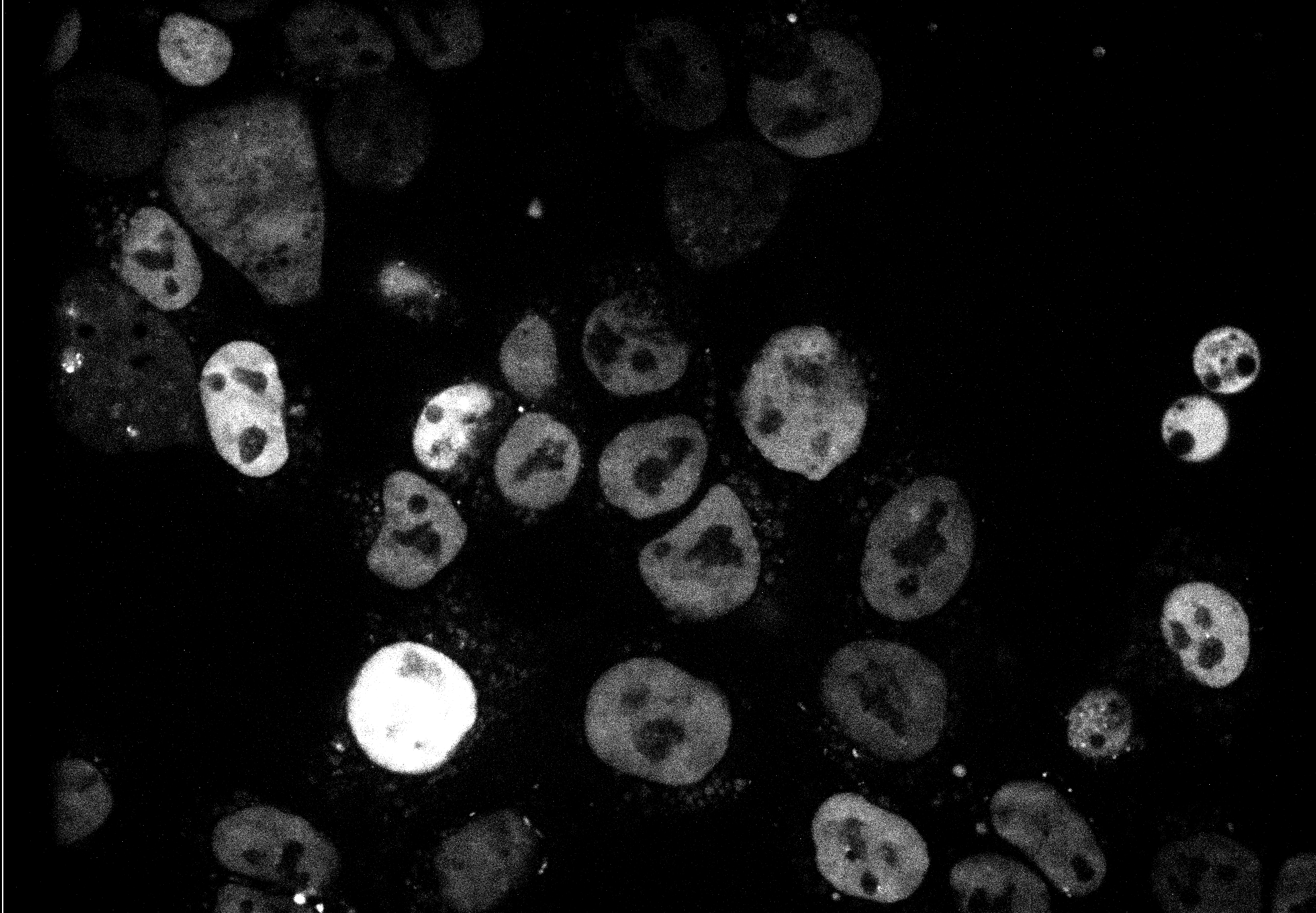

Supplement: Supplementary file 5 — Source Data for Figure 1 [file EMBR-24-e57677-s009.zip › Source data for Fig 1/EMBOR-2023-57677V2_SourceDataForFigure1D_DONSON-GFP plus p97 inhibitor.tif]

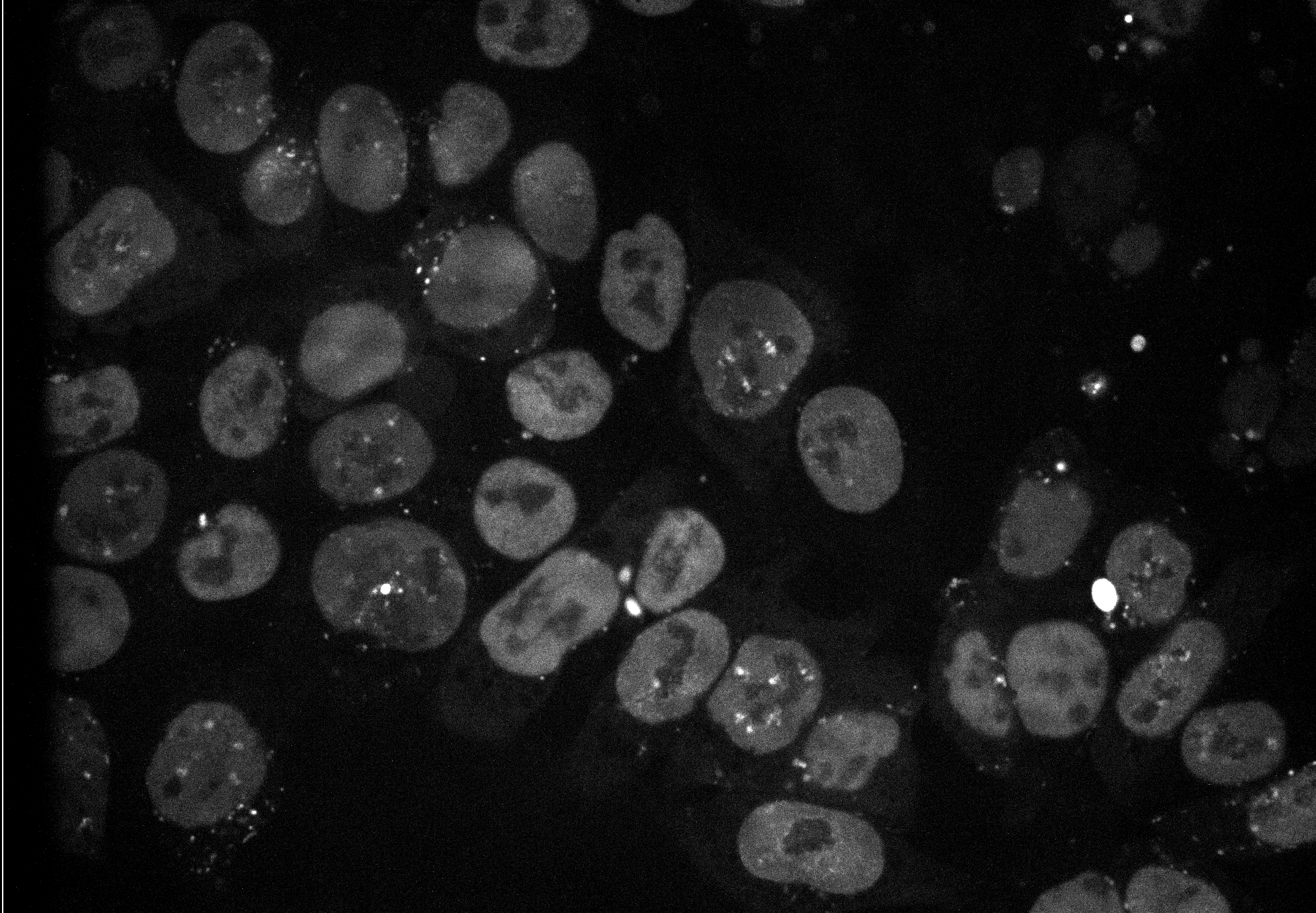

Supplement: Supplementary file 5 — Source Data for Figure 1 [file EMBR-24-e57677-s009.zip › Source data for Fig 1/EMBOR-2023-57677V2_SourceDataForFigure1D_mCherry-PSF1.tif]

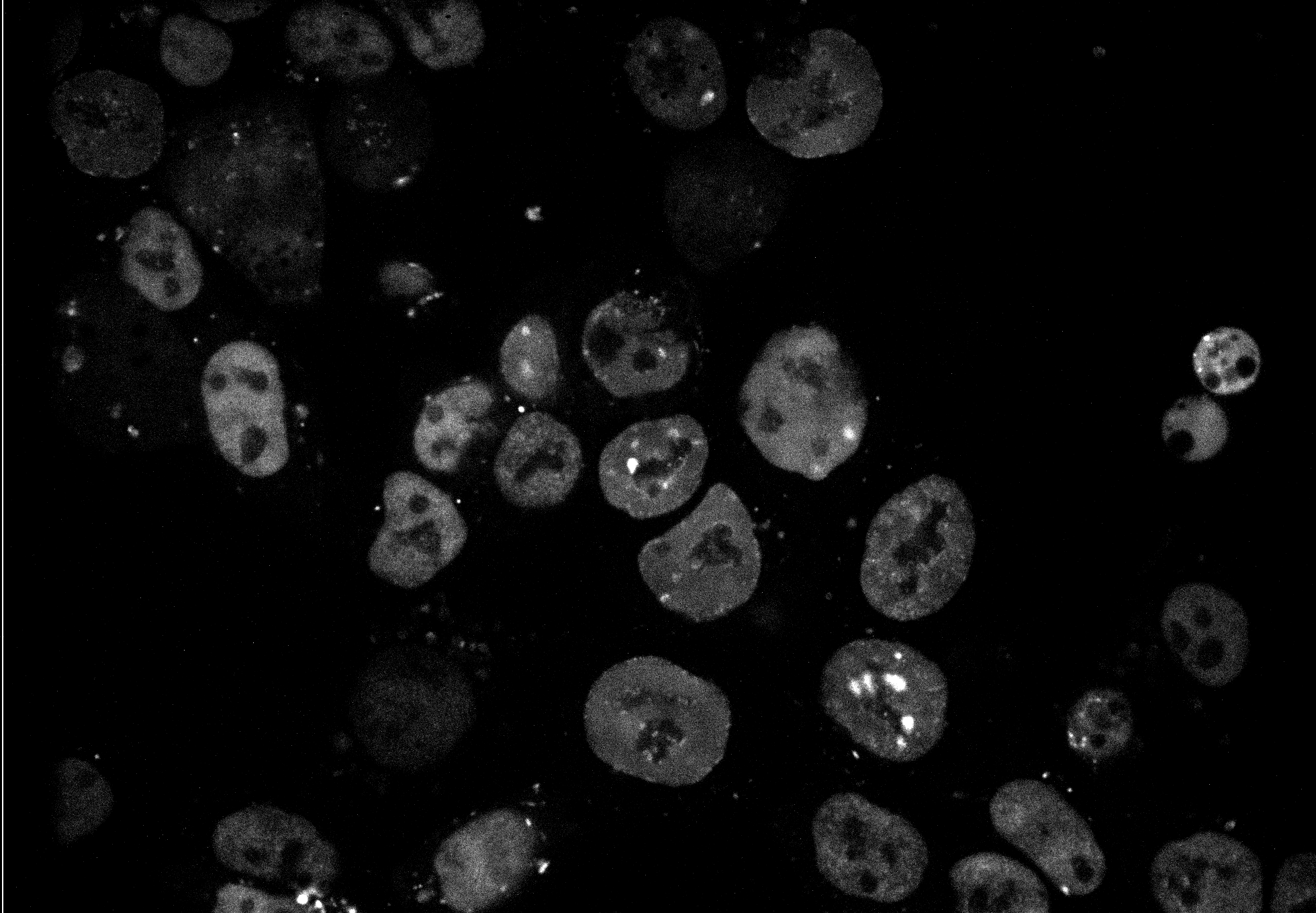

Supplement: Supplementary file 5 — Source Data for Figure 1 [file EMBR-24-e57677-s009.zip › Source data for Fig 1/EMBOR-2023-57677V2_SourceDataForFigure1D_mCherry-PSF1 plus p97 inhibitor.tif]
