# Supplementary material for: DONSON is required for CMG helicase assembly in the mammalian cell cycle
Source: EMBO Rep. 2023 Oct 2;24(11):e57677. doi: 10.15252/embr.202357677 (PMC10626419; doi:10.15252/embr.202357677)
Supplement: Supplementary file 5 — Source Data for Figure 1 [file EMBR-24-e57677-s009.zip › Source data for Fig 1/EMBOR-2023-57677V2_SourceDataForFigure1D_Read Me_1D.pdf]

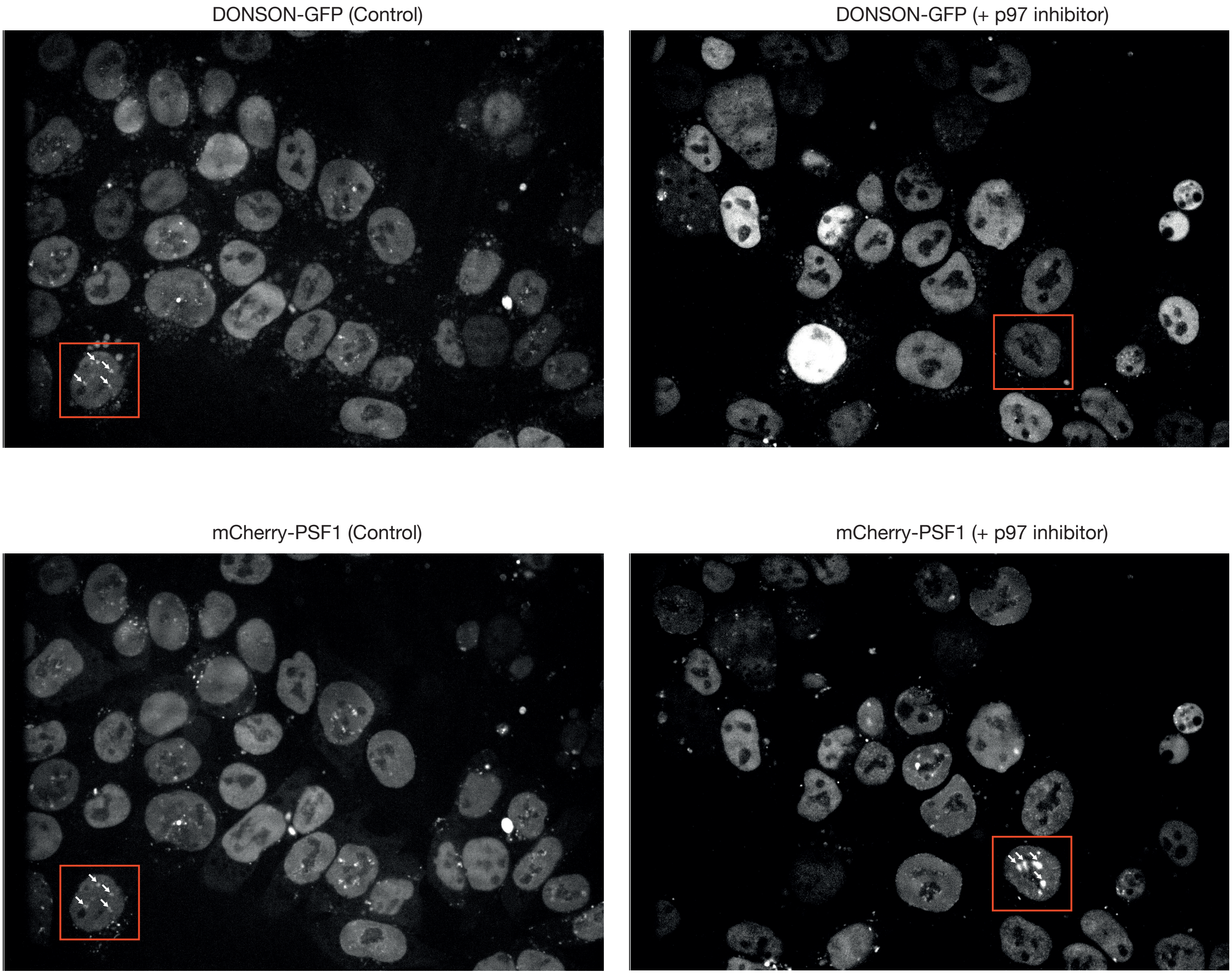


**Source data for Figure 1D.**
Micrographa with red boxes indicating the areas cropped in the Figure.
