# Supplementary material for: DONSON is required for CMG helicase assembly in the mammalian cell cycle
Source: EMBO Rep. 2023 Oct 2;24(11):e57677. doi: 10.15252/embr.202357677 (PMC10626419; doi:10.15252/embr.202357677)
Supplement: Supplementary file 9 [file EMBR-24-e57677-s008.pdf]

EMBO reports

DONSON is essential for CMG helicase assembly in the mammalian cell cycle

Cecile Evrin, Vanesa Alvarez, Johanna Ainsworth, Ryo Fujisawa, Constance Alabert, and Karim Labib
DOI: 10.15252/embr.202357677

Corresponding author(s): Karim Labib (k.p.m.labib@dundee.ac.uk) , Constance Alabert (calabert@dundee.ac.uk)

Review Timeline:

Pre-submission Date:	19th Jun 23
Pre-submission Editorial Decision:	21st Jun 23
Revision Received:	16th Aug 23
Editorial Decision:	4th Sep 23
Revision Received:	15th Sep 23
Accepted:	19th Sep 23

Editor: Esther Schnapp / Deniz Senyilmaz Tiebe

Transaction Report:

(Note: With the exception of the correction of typographical or spelling errors that could be a source of ambiguity, letters and reports are not edited. Depending on transfer agreements, referee reports obtained elsewhere may or may not be included in this compilation. Referee reports are anonymous unless the Referee chooses to sign their reports.)

Pre-submission Editorial Decision **21st Jun 2023**

Dear Karim,

Thank you for enquiring about the potential suitability of your manuscript for EMBO reports. I have now looked at your related ms as well, and discussed all with my colleagues here. We think that your ms would be a good contribution to our scientific reports section, and I will be happy to send it out for peer-review, and to fast-track its handling.

In case we can accept your manuscript for publication, it would be good to coordinate the online publication date with your related ms. In case the referees ask for major revisions that will significantly delay the publication of your EMBO reports paper, a better solution might be to publish your study in Life Science Alliance, for example. We can discuss this further when we will have received all referee comments.

Best wishes,
Esther

Esther Schnapp, PhD
Senior Editor
EMBO reports

To submit your manuscript, please follow this link.

Link Not Available

Dear Prof. Labib,

Thank you for the submission of your research manuscript to our journal, which was now seen by three referees, whose reports are copied below.

My colleague Esther is currently out of office. Therefore, I stepped in as the secondary handling editor of your manuscript.

We concur with the referees that the proposed role of DONSON in CMG helicase assembly in mammalian cells is very interesting. However, the referees raise some minor concerns that need to be addressed for publication here.

Given these positive recommendations, we would like to invite you to submit a revised manuscript. Please address all referee concerns in a complete point-by-point response.

Given the nature of the referee concerns, we believe two weeks are sufficient for the revision. Please let me know if you are unable to meet this deadline.

Also, please address the editorial points below.

- Please rename the 'Conflict of Interests' section as 'Disclosure & Competing Interests Statement'.
- We note that a response for cell D49 is currently missing in the Author Checklist.
- Please make sure that the funding information is also complete in the manuscript submission system (the Japan Society for the Promotion of Science (JSPS) Overseas Research Fellowship 202160572 to RF is currently missing).
- Please upload the main figures and the EV figures as one file per figure.
- The Appendix figure labels should be removed from the ms as they are already provided with the figures and need to be part of the Appendix file
- We note that Appendix Data S1 is source data, therefore please resubmit it as source data as one file per figure.
- We note that the Appendix file is missing a Table of Contents (please see https://www.embopress.org/page/journal/14693178/authorguide#expandedview)
- Appendix Table S1 Reagent table needs to be resubmitted either in Word or Excel format.
- At EMBO Press, we ask the authors to provide the source data that were used to generate the main figures. We note that currently source data for only Fig 2C, Fig 4A, Fig 4B and Fig 4C were submitted. Our source data coordinator will be in contact shortly with details.
- Papers published in EMBO Reports include a 'synopsis' and 'bullet points' to further enhance discoverability. Both are displayed on the html version of the paper and are freely accessible to all readers. The synopsis includes a short standfirst summarizing the study in 1 or 2 sentences (max 35 words) that summarize the paper and are provided by the authors and streamlined by the handling editor. I would therefore ask you to include your synopsis blurb and 3-5 bullet points listing the key experimental findings.
- In addition, please provide an image for the synopsis. This image should provide a rapid overview of the question addressed in the study but still needs to be kept fairly modest since the image size should be 550 pixels wide and 300-600 pixels tall.
- Our production/data editors have asked you to clarify several points in the figure legends (see attached document). Please incorporate these changes in the attached word document and return it with track changes activated.

Thank you again for giving us to consider your manuscript for EMBO Reports, I look forward to your minor revision.

Kind regards,

Deniz Senyilmaz Tiebe

--
Deniz Senyilmaz Tiebe, PhD
Editor
EMBO Reports

Referee #1:

1. Does this manuscript report a single key finding? YES
DONSON is required for CMG helicase assembly in mammalian cells.

2. Is the reported work of significance (YES), or does it describe a confirmatory finding or one that has already been documented using other methods or in other organisms etc (NO)?
The work describes something that has been recently published for other organisms, but the other publications are very recent,

and the current work is certainly of significance.

3. Is it of general interest to the molecular biology community? YES/NO
YES, the topic is very rapidly developing, and the paper addresses a key fundamental question in the field of replication initiation.

4. Is the single major finding robustly documented using independent lines of experimental evidence (YES), or is it really just a preliminary report requiring significant further data to become convincing, and thus more suited to a longer¬ format article (NO)?
YES, the finding is shown using both biochemical and imaging approaches.

The manuscript by Evrin et al. describes DONSON as a key element in the assembly of the CMG helicase during replication initiation in mammalian cells. The employed CMG purification approach provides robust evidence for their conclusions, convincingly showing that DONSON depletion results in failure to assemble CMG complexes. I believe the paper should be published in EMBO reports after minor revisions:

1. On Figure 1B the level of DONSON in the BromoTag-DONSON cells appears to be significantly lower than that in the control cells (at 0 hours) - compared to the Psf3 which appears to be very even. On Figure 3A this difference is not really noticeable. How could that be?

2. The effects of genetic modifications (tagging of DONSON and PSF1) should be clarified, did they affect cell growth and/or replication speed in any way?

3. The level of DONSON depletion at various timepoints should be quantified to better understand the efficiency of DONSON depletion.

4. DONSON appears to be fully depleted in BromoTag-DONSON cells within 2h after AGB1 addition. However, 6h (3C and 3A) and even 12h (3A) after AGB1 addition, some CMG complexes appear to still be present (3A and 3C) - the signal is clearly higher than that for the mitotic cells. The vast majority of CMGs assembled before DONSON depletion should be disassembled in that time, in my estimation, unless there is some replication stress/delay. And for the synchronized cells in 3C they just should not assemble at all. The authors should discuss possible explanations for this observation. Are some CMGs assembled independently of DONSON? Is DONSON not fully depleted? If the leftover CMG level is negligible, maybe a quantification and comparison could help.

Referee #2:

Recent work led by the Labib, Walter, Jackson and Tanaka labs has identified DONSON in a range of metazoan systems as a replication initiation factor that is required for assembly of the replicative CMG helicase. In this work, the Labib and Alabert labs extend this work by showing that DONSON is required for CMG assembly and replication initiation in mouse ES cells. Whilst the novelty is somewhat reduced given the recent flurry of papers on the topic, experiments have been very well executed, are well controlled and the manuscript is very well written. The paper also develops tools such as Bromo-tagged DONSON, which can be inducibly degraded and may prove to be a useful tool for further work on the subject. The manuscript makes a valid contribution to the field and should be published in EMBO reports.

Minor points.
1. Do the authors have an explanation for why DONSON colocalises with mCherry-PSF1 in the absence but not presence of p97 inhibitor in figure 1d? This doesn't seem to fit with Figs 1a and b, which show that DONSON can coIP with CMG under these conditions. Does this somehow relate to the fact that they are specifically analysing heterochromatin here?

2. Degradation of PSF1 or DONSON in Fig. 4a, seems to have only a minor effect on chromatin-bound MCM2 signal. If the decrease in the MCM2 signal comes from MCM unloading in response to replication termination, wouldn't a larger fraction of MCM2 be expected to be retained on chromatin without efficient CMG assembly (i.e. in the absence of DONSON or PSF1?).

The main point of the figure - that DONSON is required for chromatin association of PSF1 and SLD5 is clear.

Referee #3:

Evrin et al describe that DONSON is necessary for the assembly of the CMG in the mouse embryonic stem cells. They show that DONSON interacts directly with the CMG components in vitro (without specifically identifying the component)and is present transiently on chromatin. It is dispensable for loading the MCMs onto the chromatin, but without it, neither CDC45 nor the GINS

is present on chromatin in replicating cells. The data are convincing, and although the mechanism in not as fully explored as in the recently published manuscript from the Walter group (Lim et al 2023), it does report on a novel function of DONSON in the mammalian system. Minor changes are suggested for publication.

The CDC45 patterns in QIBC experiments are slightly different between BromoTag Donson and BromoTag-PSF1 cells. Do the authors believe that this is due to incomplete degradation of the Donson protein? In light of the other manuscript, it is likely that the loss of the CDC45 that they see in Figure 4B is secondary to loss of the GINS components. They might want to comment on that.

Technical comments:
The expression of Bromo-Tag_DONSON seems very low in comparison with levels of endogenous proteins. Why is that? I the locus otherwise intact? I appreciate that the EdU intensity seems the same, but is there a growth defect?

Figure 2D- the Y axis are not indicated, I would want to be sure that the axis was the same in all histograms

Minor comments:
Figure 1A. Please Indicate that the + are p97 as in Fig1B and include in the legend that CB-5083 is the p97 inhibitor
Figure 2 -Please define STLC in the Figure legend. They might also indicate what AGB1 is doing. It might be helpful for those who look only at the figures and not the full text of the paper.

-The sentence in lanes 226-232 is difficult to read with so many parentheses. It makes sense and is consistent with the data but just cumbersome to read. Authors might want to simplify it.

Reply to editorial points (our replies in red):

- "***Please rename the 'Conflict of Interests' section as 'Disclosure & Competing Interests Statement***".
Done – see line 640.

- "***We note that a response for cell D49 is currently missing in the Author Checklist.***"
Done

- "***Please make sure that the funding information is also complete in the manuscript submission system (the Japan Society for the Promotion of Science (JSPS) Overseas Research Fellowship 202160572 to RF is currently missing).***"
Done – see line 636.

- "***Please upload the main figures and the EV figures as one file per figure.***"
We will submit separate eps files for each figure and EV figure.

- "***The Appendix figure labels should be removed from the ms as they are already provided with the figures and need to be part of the Appendix file.***"
Done

- "***We note that Appendix Data S1 is source data, therefore please resubmit it as source data as one file per figure***".
Done

- "***We note that the Appendix file is missing a Table of Contents (please see https://www.embopress.org/page/journal/14693178/authorguide#expandedview)***".
Done

- "***Appendix Table S1 Reagent table needs to be resubmitted either in Word or Excel format.***"
We consulted with Esther Schnapp, who advised us to leave this as a pdf within the Appendix.

- "***At EMBO Press, we ask the authors to provide the source data that were used to generate the main figures. We note that currently source data for only Fig 2C, Fig 4A, Fig 4B and Fig 4C were submitted. Our source data coordinator will be in contact shortly with details.***"
Done - we now provide Source Data for Fig 1-4 and EV2-5 (the latter in a separate zipped file).

- "***Papers published in EMBO Reports include a 'synopsis' and 'bullet points' to further enhance discoverability. Both are displayed on the html version of the paper and are freely accessible to all readers. The synopsis includes a short standfirst summarizing the study in 1 or 2 sentences (max 35 words) that summarize the paper and are provided by the authors and streamlined by the***

handling editor. I would therefore ask you to include your synopsis blurb and 3-5 bullet points listing the key experimental findings."
Done

- "***In addition, please provide an image for the synopsis. This image should provide a rapid overview of the question addressed in the study but still needs to be kept fairly modest since the image size should be 550 pixels wide and 300-600 pixels tall.***"
Done

- "***Our production/data editors have asked you to clarify several points in the figure legends (see attached document). Please incorporate these changes in the attached word document and return it with track changes activated.***"
Done – we made the requested changes directly within Fig. EV1B+D (we just had to explain what an asterisk referred to).

Reply to Referee's points (our replies in red):

Referee #1:

"***1. Does this manuscript report a single key finding?***
YES
DONSON is required for CMG helicase assembly in mammalian cells.
2. Is the reported work of significance (YES), or does it describe a confirmatory finding or one that has already been documented using other methods or in other organisms etc (NO)? The work describes something that has been recently published for other organisms, but the other publications are very recent, and the current work is certainly of significance.
3. Is it of general interest to the molecular biology community? (YES/NO) YES, the topic is very rapidly developing, and the paper addresses a key fundamental question in the field of replication initiation.
4. Is the single major finding robustly documented using independent lines of experimental evidence (YES), or is it really just a preliminary report requiring significant further data to become convincing, and thus more suited to a longer¬ format article (NO)? YES, the finding is shown using both biochemical and imaging approaches. The manuscript by Evrin et al. describes DONSON as a key element in the assembly of the CMG helicase during replication initiation in mammalian cells. The employed CMG purification approach provides robust evidence for their conclusions, convincingly showing that DONSON depletion results in failure to assemble CMG complexes. I believe the paper should be published in EMBO reports after minor revisions:"

"***1. On Figure 2B the level of DONSON in the BromoTag-DONSON cells appears to be significantly lower than that in the control cells (at 0 hours) - compared to the Psf3 which appears to be very even. On Figure 3A this difference is not really noticeable. How could that be?***"
This is down to the different ways of making cell extracts in the two cases - the difference in level between control and BromoTag-DONSON is more apparent when we make denatured extracts (Figure 2B), compared to native extracts (Figure 3A). This suggests that the solubility of DONSON is limiting in our native extracts, so that some protein is lost when we centrifuge the extracts (not such an issue for BromoTag-DONSON that is expressed to a lower level).
For clarity, we now make clear in Materials and Methods (line 459 and line 470) which extract method was used for which experiments.

2. "***The effects of genetic modifications (tagging of DONSON and PSF1) should be clarified, did they affect cell growth and/or replication speed in any way?***"
Tagging DONSON or PSF1 did not affect cell growth in (e.g. Fig 2A for BromoTag-DONSON; Fig EV5C for BromoTag-PSF1).
Moreover, the quantitative imaging based cytometry data in Fig 4 show that loading of MCM / CDC45 / GINS were comparable in control and tagged cells, prior to depletion of BromoTag-PSF1 or BromoTag-DONSON (0h timepoint).
Furthermore, EdU incorporation data in Fig2C show that DNA replication is very similar in BromoTag-DONSON and control cells (0h timepoint).

This is now discussed at the end of the methods section describing the generation of mouse ES cells with tagged PSF1 or DONSON (lines 390-392).

3. "***The level of DONSON depletion at various timepoints should be quantified to better understand the efficiency of DONSON depletion.***"
Quantification is now provided on line 182 (degradation did not improve beyond 2 hours). As with any depletion experiment, by any method, depletion will never be 100% and it's always possible (and indeed likely) that the remaining small amount of protein can provide some residual function.

4. "***DONSON appears to be fully depleted in BromoTag-DONSON cells within 2h after AGB1 addition. However, 6h (3C and 3A) and even 12h (3A) after AGB1 addition, some CMG complexes appear to still be present (3A and 3C) - the signal is clearly higher than that for the mitotic cells. The vast majority of CMGs assembled before DONSON depletion should be disassembled in that time, in my estimation, unless there is some replication stress/delay. And for the synchronized cells in 3C they just should not assemble at all. The authors should discuss possible explanations for this observation. Are some CMGs assembled independently of DONSON? Is DONSON not fully depleted? If the leftover CMG level is negligible, maybe a quantification and comparison could help.***"
As discussed above, degradation of BromoTag-DONSON is very good (~ 95%) but not perfect, so residual protein provides one potential explanation for why depletion of CMG is incomplete.
In addition, we suspect that DONSON is not only required for CMG assembly, but also for CMG activation. In that case, some newly assembled CMG complexes might be blocked in an inactive state when DONSON is degraded, also helping to explain the data in Fig. 3.
We now discuss both possibilities in the revised manuscript (lines 222 – 225). Nevertheless, it's clear that depletion of DONSON leads to a dramatic loss of CMG, and a failure to accumulate CMG during entry into S-phase. Moreover, the data in Fig. 4 show clearly that the defect in CMG assembly is after the loading of MCM2-7. In other words, DONSON is required for stable association of GINS and CDC45 with MCM2-7 to form the CMG complex.

Referee #2:
"***Recent work led by the Labib, Walter, Jackson and Tanaka labs has identified DONSON in a range of metazoan systems as a replication initiation factor that is required for assembly of the replicative CMG helicase. In this work, the Labib and Alabert labs extend this work by showing that DONSON is required for CMG assembly and replication initiation in mouse ES cells. Whilst the novelty is somewhat reduced given the recent flurry of papers on the topic, experiments have been very well executed, are well controlled and the manuscript is very well written. The paper also develops tools such as Bromo-tagged DONSON, which can be inducibly degraded and may prove to be a useful tool for further work on the subject. The manuscript makes a valid contribution to the field and should be published in EMBO reports.***"
Minor points.
1. "***Do the authors have an explanation for why DONSON colocalises with mCherry-PSF1 in the absence but not presence of p97 inhibitor in figure 1d?***

This doesn't seem to fit with Figs 1a and b, which show that DONSON can coIP with CMG under these conditions. Does this somehow relate to the fact that they are specifically analysing heterochromatin here?"

This fits beautifully with the fact that DONSON is an initiation factor, rather than a replisome component. DONSON interacts with CMG, but it only does so in a very dynamic way, contrasting with replisome components that associate more stably with CMG. We now discuss this interesting point on lines 282-284.

2. "***Degradation of PSF1 or DONSON in Fig. 4a, seems to have only a minor effect on chromatin-bound MCM2 signal. If the decrease in the MCM2 signal comes from MCM unloading in response to replication termination, wouldn't a larger fraction of MCM2 be expected to be retained on chromatin without efficient CMG assembly (i.e. in the absence of DONSON or PSF1?).***"

There is perhaps some confusion here – it is indeed true that degradation of PSF1 or DONSON does not reduce chromatin-bound MCM2, compared to GINS and CDC45 (Fig 4B-C), but this was the whole point of the experiment. DONSON is dispensable for loading of MCM2-7 onto chromatin in G1 phase, and instead is required for CMG assembly during S-phase. These data identify the step at which DONSON functions during DNA replication initiation (CMG assembly during S-phase).

"***The main point of the figure - that DONSON is required for chromatin association of PSF1 and SLD5 is clear.***"

Yes!

Referee #3:

"***Evrin et al describe that DONSON is necessary for the assembly of the CMG in the mouse embryonic stem cells. They show that DONSON interacts directly with the CMG components in vitro (without specifically identifying the component) and is present transiently on chromatin. It is dispensable for loading the MCMs onto the chromatin, but without it, neither CDC45 nor the GINS is present on chromatin in replicating cells. The data are convincing, and although the mechanism in not as fully explored as in the recently published manuscript from the Walter group (Lim et al 2023), it does report on a novel function of DONSON in the mammalian system. Minor changes are suggested for publication.***"

"***The CDC45 patterns in QIBC experiments are slightly different between BromoTag Donson and BromoTag-PSF1 cells. Do the authors believe that this is due to incomplete degradation of the Donson protein?***"

Yes – as discussed above, depletion of degron-tagged proteins is unlikely ever to be complete and whether residual protein can still supply function must be determined empirically in each case. As discussed on line 182, we now present data showing that ~95% of BromoTag-DONSON is degraded, and although this blocks DNA replication and CMG helicase assembly, we suspect that the remaining ~5% still provides some measure of residual function.

Degradation of BromoTag-PSF1 produces a similar but slightly stronger phenotype, compared with degradation of BromoTag-DONSON. We now present new data to explain this difference. PSF1 functions in a tight complex (GINS) with PSF2, PSF3 and SLD5, and we show that degradation of BromoTag-PSF1 destabilises PSF3 (Fig EV5D, cited on lines 260-261), which helps to inactivate GINS function. This is

consistent with previous data showing that PSF3 is only soluble when part of the GINS complex.

"*In light of the other manuscript, it is likely that the loss of the CDC45 that they see in Figure 4B is secondary to loss of the GINS components. They might want to comment on that.*"
We now discuss this point on lines 316-319.

Technical comments:
"***The expression of Bromo-Tag_DONSON seems very low in comparison with levels of endogenous proteins. Why is that? I the locus otherwise intact?"***
This is likely to be a consequence of N-terminal tagging with BromoTag, since we found that C-terminal tagging of DONSON with BromoTag did not reduce expression. In practice, this was a useful phenomenon, as the lower expression helps with functional depletion of DONSON to a level that blocks DNA replication.

"***I appreciate that the EdU intensity seems the same, but is there a growth defect?***"
Tagging DONSON did not affect cell growth in (Fig 2A for BromoTag-DONSON). Moreover, the quantitative imaging-based cytometry data in Fig 4 show that loading of MCM / CDC45 / GINS were comparable in control and BromoTag-Donson cells, prior to depletion of BromoTag-DONSON (0h timepoint).
As the referee notes, EdU incorporation data in Fig2C show that DNA replication is very similar in BromoTag-DONSON and control cells (0h timepoint).

"***Figure 2D- the Y axis are not indicated, I would want to be sure that the axis was the same in all histograms.***"
We now include a label for the Y-axis (Cell Number) and can confirm that the axis is the same in all histograms.

Minor comments:
"***Figure 1A. Please Indicate that the + are p97 as in Fig1B and include in the legend that CB-5083 is the p97 inhibitor***"
Thanks for pointing this out – we have now corrected Fig1A and explained this point in the figure legend (lines 777-778).

"Figure 2 -Please define STLC in the Figure legend. They might also indicate what AGB1 is doing. It might be helpful for those who look only at the figures and not the full text of the paper."
We now explain in the figure legend (lines 804-805) that STLC blocks cells in mitosis by inhibiting the kinesin Eg5. The acronym STLC is defined at its first usage in the main text (line 194).
We also explain the action of AGB1 in the figure legend on lines 794-795.

"***The sentence in lanes 226-232 is difficult to read with so many parentheses. It makes sense and is consistent with the data but just cumbersome to read. Authors might want to simplify it.***"
Thanks for pointing this out – we have now split this sentence into two and rephrased for greater clarity (lines 230-236).

Prof. Karim Labib
University of Dundee
MRC Protein Phosphorylation and Ubiquitylation Unit
Sir James Black Centre
Dow Street
Dundee DD1 5EH
United Kingdom

Dear Karim,

I am very pleased to accept your manuscript for publication in the next available issue of EMBO reports. Thank you for your contribution to our journal.

At the end of this email I include important information about how to proceed. Please ensure that you take the time to read the information and complete and return the necessary forms to allow us to publish your manuscript as quickly as possible.

As part of the EMBO publication's Transparent Editorial Process, EMBO reports publishes online a Review Process File to accompany accepted manuscripts. As you are aware, this File will be published in conjunction with your paper and will include the referee reports, your point-by-point response and all pertinent correspondence relating to the manuscript.

If you do NOT want this File to be published, please inform the editorial office within 2 days, if you have not done so already, otherwise the File will be published by default [contact: emboreports@embo.org]. If you do opt out, the Review Process File link will point to the following statement: "No Review Process File is available with this article, as the authors have chosen not to make the review process public in this case."

Should you be planning a Press Release on your article, please get in contact with emboreports@wiley.com as early as possible, in order to coordinate publication and release dates.

Thank you again for your contribution to EMBO reports and congratulations on a successful publication. Please consider us again in the future for your most exciting work.

Best regards,
Esther

Esther Schnapp, PhD
Senior Editor
EMBO reports

****

THINGS TO DO NOW:

Please note that you will be contacted by Wiley Author Services to complete licensing and payment information. The required 'Page Charges Authorization Form' is available here: https://www.embopress.org/pb-assets/embo-site/er_apc.pdf - please download and complete the form and return to embopressproduction@wiley.com

EMBO Press participates in many Publish and Read agreements that allow authors to publish Open Access with reduced/no publication charges. Check your eligibility: https://authorservices.wiley.com/author-resources/Journal-Authors/open-access/affiliation-policies-payments/index.html

You will receive proofs by e-mail approximately 2-3 weeks after all relevant files have been sent to our Production Office; you should return your corrections within 2 days of receiving the proofs.

Please inform us if there is likely to be any difficulty in reaching you at the above address at that time. Failure to meet our deadlines may result in a delay of publication, or publication without your corrections.

All further communications concerning your paper should quote reference number EMBOR-2023-57677V2 and be addressed to emboreports@wiley.com.

Should you be planning a Press Release on your article, please get in contact with emboreports@wiley.com as early as possible, in order to coordinate publication and release dates.

EMBO Press Author Checklist

Corresponding Author Name: KARIM LABIB	
Journal Submitted to: EMBO Reports	
Manuscript Number: EMBOR-2023-57677V1	

USEFUL LINKS FOR COMPLETING THIS FORM
The EMBO Journal - Author Guidelines
EMBO Reports - Author Guidelines
Molecular Systems Biology - Author Guidelines
EMBO Molecular Medicine - Author Guidelines

Reporting Checklist for Life Science Articles (updated January
This checklist is adapted from Materials Design Analysis Reporting (MDAR) Checklist for Authors. MDAR establishes a minimum set of requirements in transparent reporting in the life sciences (see Statement of Task: 10.31222/osf.io/9sm4x). Please follow the journal's guidelines in preparing your manuscript.
Please note that a copy of this checklist will be published alongside your article.

Abridged guidelines for figures
1. Data
The data shown in figures should satisfy the following conditions:
- the data were obtained and processed according to the field's best practice and are presented to reflect the results of the experiments in an accurate and unbiased manner.
- ideally, figure panels should include only measurements that are directly comparable to each other and obtained with the same assay.
- plots include clearly labeled error bars for independent experiments and sample sizes. Unless justified, error bars should not be shown for technical
- if n<5, the individual data points from each experiment should be plotted. Any statistical test employed should be justified.
- Source Data should be included to report the data underlying figures according to the guidelines set out in the authorship guidelines on Data

2. Captions
Each figure caption should contain the following information, for each panel where they are relevant:
- a specification of the experimental system investigated (eg cell line, species name).
- the assay(s) and method(s) used to carry out the reported observations and measurements.
- an explicit mention of the biological and chemical entity(ies) that are being measured.
- an explicit mention of the biological and chemical entity(ies) that are altered/varied/perturbed in a controlled manner.
- the exact sample size (n) for each experimental group/condition, given as a number, not a range;
- a description of the sample collection allowing the reader to understand whether the samples represent technical or biological replicates (including how many animals, litters, cultures, etc.).
- a statement of how many times the experiment shown was independently replicated in the laboratory.
- definitions of statistical methods and measures:
  - common tests, such as t-test (please specify whether paired vs. unpaired), simple χ2 tests, Wilcoxon and Mann-Whitney tests, can be unambiguously identified by name only, but more complex techniques should be described in the methods section;
  - are tests one-sided or two-sided?
  - are there adjustments for multiple comparisons?
  - exact statistical test results, e.g., P values = x but not P values < x;
  - definition of 'center values' as median or average;
  - definition of error bars as s.d. or s.e.m.

Please complete ALL of the questions below.
Select "Not Applicable" only when the requested information is not relevant for your study.

Materials

Newly Created Materials	Information included in the manuscript?	In which section is the information available? (Reagents and Tools Table, Materials and Methods, Figures, Data Availability Section)
New materials and reagents need to be available; do any restrictions apply?	Not Applicable	Appendix Table S1, Materials and Methods

Antibodies	Information included in the manuscript?	In which section is the information available? (Reagents and Tools Table, Materials and Methods, Figures, Data Availability Section)
For **antibodies** provide the following information:<br>- Commercial antibodies: RRID (if possible) or supplier name, catalogue number and or/clone number<br>- Non-commercial: RRID or citation	Yes	Appendix Table S1

DNA and RNA sequences	Information included in the manuscript?	In which section is the information available? (Reagents and Tools Table, Materials and Methods, Figures, Data Availability Section)
**Short novel DNA or RNA including primers, probes**: provide the sequences.	Yes	Appendix Table S1

Cell materials	Information included in the manuscript?	In which section is the information available? (Reagents and Tools Table, Materials and Methods, Figures, Data Availability Section)
**Cell lines:** Provide species information, strain. Provide accession number in repository **OR** supplier name, catalog number, clone number, and/**OR** RRID.	Yes	Appendix Table S1, Materials and Methods
**Primary cultures:** Provide species, strain, sex of origin, genetic modification status.	Not Applicable	
Report if the cell lines were recently **authenticated** (e.g., by STR profiling) and tested for mycoplasma contamination.	Yes	DNA sequence analysis of multiple loci; regular mycoplasma testing.

Experimental animals	Information included in the manuscript?	In which section is the information available? (Reagents and Tools Table, Materials and Methods, Figures, Data Availability Section)
**Laboratory animals or Model organisms:** Provide species, strain, sex, age, genetic modification status. Provide accession number in repository **OR** supplier name, catalog number, clone number, **OR** RRID.	Not Applicable	
**Animal observed in or captured from the field:** Provide species, sex, and age where possible**.**	Not Applicable	
Please detail **housing and husbandry conditions**.	Not Applicable	

Plants and microbes	Information included in the manuscript?	In which section is the information available? (Reagents and Tools Table, Materials and Methods, Figures, Data Availability Section)
**Plants:** provide species and strain, ecotype and cultivar where relevant, unique accession number if available, and source (including location for collected wild specimens).	Not Applicable	
**Microbes:** provide species and strain, unique accession number if available, and source.	Yes	Appendix Table S1, Materials and Methods

Human research participants	Information included in the manuscript?	In which section is the information available? (Reagents and Tools Table, Materials and Methods, Figures, Data Availability Section)
If collected and within the bounds of privacy constraints report on age, sex and gender or ethnicity for all study participants.	Not Applicable	

Core facilities	Information included in the manuscript?	In which section is the information available? (Reagents and Tools Table, Materials and Methods, Figures, Data Availability Section)
If your work benefited from core facilities, was their service mentioned in the acknowledgments section?	Yes	Materials and Methods

Design

Study protocol	Information included in the manuscript?	In which section is the information available? (Reagents and Tools Table, Materials and Methods, Figures, Data Availability Section)
If study protocol has been **pre-registered, provide DOI in the manuscript**. For clinical trials, provide the trial registration number **OR** cite DOI.	Not Applicable	
Report the **clinical trial registration number** (at ClinicalTrials.gov or equivalent), where applicable.	Not Applicable	

Laboratory protocol	Information included in the manuscript?	In which section is the information available? (Reagents and Tools Table, Materials and Methods, Figures, Data Availability Section)
Provide DOI OR other citation details if **external detailed step-by-step protocols** are available.	Not Applicable	

Experimental study design and statistics	Information included in the manuscript?	In which section is the information available? (Reagents and Tools Table, Materials and Methods, Figures, Data Availability Section)
Include a statement about **sample size** estimate even if no statistical methods were used.	Yes	Materials and Methods
Were any steps taken to minimize the effects of subjective bias when allocating animals/samples to treatment (e.g. **randomization procedure**)? If yes, have they been described?	Not Applicable	
Include a statement about **blinding** even if no blinding was done.	Not Applicable	
Describe **inclusion/exclusion criteria** if samples or animals were excluded from the analysis. Were the criteria pre-established?<br><br>If sample or data points were omitted from analysis, report if this was due to attrition or intentional exclusion and provide justification.	Not Applicable	
For every figure, are **statistical tests** justified as appropriate? Do the data meet the assumptions of the tests (e.g., normal distribution)? Describe any methods used to assess it. Is there an estimate of variation within each group of data? Is the variance similar between the groups that are being statistically compared?	Not Applicable	

Sample definition and in-laboratory replication	Information included in the manuscript?	In which section is the information available? (Reagents and Tools Table, Materials and Methods, Figures, Data Availability Section)
In the figure legends: state number of times the experiment was **replicated** in laboratory.	Not Applicable	
In the figure legends: define whether data describe **technical or biological replicates**.	Not Applicable	

Ethics

Ethics	Information included in the manuscript?	In which section is the information available? (Reagents and Tools Table, Materials and Methods, Figures, Data Availability Section)
Studies involving **human participants**: State details of **authority granting ethics approval** (IRB or equivalent committee(s), provide reference number for approval.	Not Applicable	
Studies involving **human participants**: Include a statement confirming that **informed consent** was obtained from all subjects and that the experiments conformed to the principles set out in the WMA Declaration of Helsinki and the Department of Health and Human Services Belmont Report.	Not Applicable	
Studies involving **human participants:** For publication of **patient photos**, include a statement confirming that consent to publish was obtained.	Not Applicable	
Studies involving experimental **animals**: State details of **authority granting ethics approval** (IRB or equivalent committee(s), provide reference number for approval. Include a statement of compliance with ethical regulations.	Not Applicable	
Studies involving **specimen and field samples:** State if relevant **permits** obtained, provide details of authority approving study; if none were required, explain why.	Not Applicable	

Dual Use Research of Concern (DURC)	Information included in the manuscript?	In which section is the information available? (Reagents and Tools Table, Materials and Methods, Figures, Data Availability Section)
Could your study fall under dual use research restrictions? Please check biosecurity documents and list of **select agents and toxins** (CDC): https://www.selectagents.gov/sat/list.htm	Not Applicable	
If you used a select agent, is the security level of the lab appropriate and reported in the manuscript?	Not Applicable	
If a study is subject to dual use research of concern regulations, is the name of the **authority granting approval and reference number** for the regulatory approval provided in the manuscript?	Not Applicable	

Reporting

The MDAR framework recommends adoption of discipline-specific guidelines, established and endorsed through community initiatives. Journals have their own policy about requiring specific guidelines and recommendations to complement MDAR.

Adherence to community standards	Information included in the manuscript?	In which section is the information available? (Reagents and Tools Table, Materials and Methods, Figures, Data Availability Section)
State if relevant guidelines or checklists (e.g., **ICMJE, MIBBI, ARRIVE, PRISMA**) have been followed or provided.	Not Applicable	
For **tumor marker prognostic studies,** we recommend that you follow the **REMARK** reporting guidelines (see link list at top right). See author guidelines, under 'Reporting Guidelines'. Please confirm you have followed these guidelines.	Not Applicable	
For **phase II and III randomized controlled trials**, please refer to the **CONSORT** flow diagram (see link list at top right) and submit the CONSORT checklist (see link list at top right) with your submission. See author guidelines, under 'Reporting Guidelines'. Please confirm you have submitted this list.	Not Applicable	

Data Availability

Data availability	Information included in the manuscript?	In which section is the information available? (Reagents and Tools Table, Materials and Methods, Figures, Data Availability Section)
Have **primary datasets** been deposited according to the journal's guidelines (see 'Data Deposition' section) and the respective accession numbers provided in the Data Availability Section?	Not Applicable	
Were **human clinical and genomic datasets** deposited in a public access-controlled repository in accordance to ethical obligations to the patients and to the applicable consent agreement?	Not Applicable	
Are **computational models** that are central and integral to a study available without restrictions in a machine-readable form? Were the relevant accession numbers or links provided?	Not Applicable	
If publicly available data were reused, provide the respective **data citations in the reference list**.	Not Applicable	
